# Quantifying the dynamic recovery of plants through stress memory and physiological attractors

**DOI:** 10.1038/s41598-026-49352-y

**Published:** 2026-04-30

**Authors:** Rashid Iqbal Khan, Mazhar Abbas, Rana Usman Zahid, Ayesha Maryam, Khurram Ziaf, Salman Mumtaz, Muhammad Azeem Sabir, Sammen Walli, Shakirullah Khan, Abdul Rauf

**Affiliations:** 1 Institute of Horticultural Sciences, University of Agriculture, Faisalabad, Pakistan; 2 Horticultural Research Institute, National Agricultural Research Center, Islamabad, Pakistan; 3 Department of Agriculture & Agribusiness Management, University of Karachi, Karachi, Pakistan; 4 National Institute of Food Science and Technology (NIFSAT), University of Agriculture, Faisalabad, Pakistan; 5 Department of Agriculture Science, Allama Iqbal Open University, Islamabad, Pakistan; 6 Department of Plant Pathology, University of Agriculture, Faisalabad, Pakistan; 7 Institute of Forest Sciences, The Islamia University of Bahawalpur, Bahawalpur, Pakistan; 8 Department of Horticulture, PMAS-Arid Agriculture University, Rawalpindi, Pakistan; 9 Department of Horticulture, Bahauddin Zakariya University, Multan, Pakistan

**Keywords:** Climate resilience, Nonlinear dynamics, Plant stress memory, Phenological synchronization, State-space trajectory, Ecology, Ecology, Physiology, Plant sciences

## Abstract

Climate change-induced weather variability poses a growing threat to global food security, yet plant resilience is still interpreted through static and reductionist models that treat stress as independent and transient. Here, we introduce a unified quantitative framework grounded in dynamical systems theory. We formalize three novel metrics: (1) the Phenological Weather Memory Index (PWMI), that quantifies exponentially decaying stress memory across developmental stages (with a decay constant α = 0.10 determined by cross‑validation); (2) the Treatment-Weather Resonance Coefficient (TWRC), which measures the alignment of agronomic interventions with favorable weather conditions; and (3) the Physiological State-Space Trajectory (PSST), which maps multi-trait plant physiology into low dimensional attractor basins. Analyzing 288 tomato plants across 24 cultivars under hot, sub-tropical conditions (mean VPD: 2.53 kPa, 65 heat days > 35 °C), we discovered that stress memory is strongly phase-dependent, remaining minimal during vegetative growth (PWMI = 0.009) but increasing sharply during reproductive phase (PWMI = 0.574). Despite the prolonged thermal stress, 97.9% of plants converged into a stable high-yield attractor basin, revealing a fundamental nonlinearity in plant performance. This convergence was driven by dynamic recovery, defined as the capacity of certain cultivars to rapidly forget the stress memory while maintaining internal physiological flexibility. Cultivars such as ‘Pony Express’, combined low PWMI with effective treatment-weather synchronization, enabling stable productivity under extreme conditions. Together, these results demonstrate that resilience is not a static trait of endurance, but an emergent property arising from temporal synchronization, rapid stress recovery and stable physiological organization. By quantifying stress “forgetting curves” and attractor dynamics, this framework provides a predictive, systems-based foundation for breeding and management strategies that prioritize dynamic recovery over stress tolerance alone.

**Supplementary Information:**

The online version contains supplementary material available at 10.1038/s41598-026-49352-y.

## Introduction

Global food security is facing an unprecedented challenge under the accelerating pace of climate change ^1^. This climate change has not only reduced crop yields but also makes them more variable across the seasons, thus making the agriculture riskier and less predictable ^2^. Meanwhile, despite decades of advanced research on crop modeling and stress physiology, our understanding of plant resilience is limited by a static, linear, and reductionist framework. The conventional crop models neglect the cumulative and history dependent behavior of plants and only focus on environmental stressors as independent, daily events having linear & instantaneous responses ^3^. Therefore, agronomic interventions practices (i.e., application of fertilizers, growth hormones, or irrigation) are typically scheduled based on calendar dates or phenological stages, ignoring the current interaction between the plant’s physiological state and the prevailing environmental conditions ^4^.

This static paradigm fails to consider several parameters of crucial importance to plant survival and productivity under stress i.e., physiological memory, non-linear state transitions and recovery dynamics. Now, it has been qualitatively accepted that plant response under stress is neither independent nor ephemeral, but contains a stress memory whereby earlier stress exposure has subsequent physiological impacts ^5^. Upon exposure to any stress (e.g., a short drought or heat spike) leaves the long-lasting impacts on physiological and molecular responses that affects the growth and final yield even after the return of favorable environmental conditions ^6^. This memory is not genetic and manifests through physiological and regulatory persistence that derives for the plant defense mechanisms. But this crucial concept has remained mostly qualitative. The plant scientists lack a strong, quantitative metric to answer some fundamental questions i.e., “How long does a plant remember a specific stress event? “Does this memory decay exponentially or linearly?” and “How does the duration of this memory vary across different growth phases and genotypes?”.

The absence of such critical metrics results in inaccurate yield predictions, sub-optimal management decisions and breeding programs continue to prioritize stress tolerance (the ability to withstand stress) rather than selecting for stress recovery (the ability to quickly “forget” the stress and resume normal function) ^7^. Under increasingly episodic climatic extremes, recovery dynamics may be more crucial as compared to tolerance itself. However, our existing modelling approach fails to capture recovery as an emergent property of physiological processes unfolding over time.

From a dynamical systems perspective, when a plant is subjected to prolonged or intense stress, its physiological state does not simply decline linearly; rather, the system can transition abruptly from a stable, high-productivity state to a low-productivity or failure state or attractor basins ^8^. Moreover, once critical threshold levels are crossed, plants may shift to irreversible lower-yield or failure states which explains why late interventions are often failed even upon the arrival of favorable environmental conditions. Yet, these non-linear transitions are rarely quantified in plant studies and plant physiological traits are still analyzed in isolation rather than a component of integrated and dynamic system.

In addition, the inconsistent efficacy of agronomic interventions under stress conditions exacerbates this problem ^9^. These variations are often attributed to differences in dosage or product quality, ignoring the crucial factor, “the timing of the intervention relative to the plant’s receptivity and the environmental conditions”. Similar interventions have been producing different outcomes depending upon their coincidence with favorable and stable environmental conditions ^10^. Therefore, to maximize the economic return and minimize the environmental footprint of high-value inputs, a framework is needed to quantify the degree of synchronization between a management action, plant physiological state and the prevailing weather patterns.

To address these interconnected gaps, we introduce a unified quantitative framework derived from dynamical systems theory that redefines plant resistance as an emergent property of memory, timing and physiological state dynamics. This novel framework integrates three complementary metrics. First, the Phenological Weather Memory Index (PWMI) quantifies cumulative stress memory using an exponential decay function that captures how the influence of past stress events diminishes over time. In the present study, the decay parameter α was selected globally using cross-validation and then applied consistently across cultivars, allowing genotype differences to emerge through PWMI values rather than cultivar-specific decay-rate estimates. Secondly, the Treatment-Weather Resonance Coefficient (TWRC) measures the capacity to synchronization between agronomic interventions and favorable, stable weather windows throughout crucial developmental stages. Third, the Physiological State-Space Trajectory (PSST) framework applies multivariate plant physiology into several state-spaces thereby enabling the identification of plants trajectory, stable attractor basins w.r.t yield outcomes and non-linear state transitions under stress.

All of these components are not independent but instead inter-connected and linked with each other. PWMI measures the plant internal memory, TWRC identifies the environmental window with treatment application and PSST tracks the system trajectories into different physiological state space. Together, all these forms a closed-loop approach which connects past stress, current interventions and future outcomes thereby enabling plant resilience to be quantified as a dynamic process of recovery and convergence rather than as a static characteristic.

To validate the above said framework, we carried out a field-based experiment with 24 tomato cultivars grown under prolonged heat stress in a hot, sub-tropical environment. In particular, we investigated whether plants have measurable, phase-dependent stress memory; whether the timing of agronomic interventions in relation to weather patterns determines their efficacy more than their type or dosage and whether plant physiology can be modeled as a dynamical system with distinct attractor basins that offer early warning signals of yield collapse. By answering these questions, our study shifts the emphasis from stress tolerance to dynamic recovery mechanics and builds a predictive, systems-based framework for comprehending and designing climate resilience crops.

## Material and methods

### Experimental design and data collection

A field-based research trial comprising twenty-four tomato genotypes (Table 1) was conducted under hot, sub-tropical conditions at the Vegetable Research Area, Institute of Horticultural Sciences (${31.4303}^{\circ }\mathbf{N},{73.0672}^{\circ }\mathbf{E}$), University of Agriculture, Faisalabad, Pakistan. The experiment was laid following Randomized Complete Block Design (RCBD) with 288 plants of 24 cultivars under four management treatments (${\boldsymbol{n}}=72$ per treatment). The daily climate data i.e., temperature, rainfall, humidity, vapor pressure deficit (VPD) and growing degree days (GDD), were recorded via an on-site weather station, while the physico chemical characteristics of the soil are summarized in Table 2. The data was recorded for physiological, biochemical, and phenological traits were measured, including antioxidant activities (SOD, POD, CAT), nutrient concentrations (N, P, K), and yield components. Superoxide dismutase (SOD), peroxidase (POD), and catalase (CAT) activities were measured spectrophotometrically following the methods of Stagner and Ppovic (2009), Liu et al*.* (2009) and Chance and Maehly, (1955) respectively. Nitrogen (N) was determined by the Kjeldahl method (Saez-Plaza et al., 2013) phosphorus (P) by the vanadomolybdate method ^32^ and potassium (K) by flame photometry as described in Estefan et al. ^33^.

**Table 1:** Physico-chemical characteristics of the sandy loam soil at the experimental site.

Soil	Units	Value	Water	Units	Value
Texture	Loam	Loam	pH	-	7.25
pH	-	8.01	Conductivity	µS cm^−1^	913
ECe	dSm^-1^	2.32	Carbonates	meq L^−1^	0.00
OM	%	0.64	Bicarbonates	meq L^−1^	0.75
N	%	0.032	Chlorides	meq L^−1^	1.63
P	mg kg^-1^	6.35	Ca + Mg	meq L^−1^	8.76
K	mg kg^-1^	128	SAR	-	1.54

ECe = Electrical Conductivity of soil extract; SAR = Sodium Adsorption Ratio; meq = milliequivalent

**Table 2:** List of twenty-four tomato genotypes used in the study.

SR. NO	NAME	TYPE	SOURCE
1	Naqeeb	Determinate OPV	AARI, Faisalabad Pakistan
2	Nadir	Determinate OPV	AARI, Faisalabad Pakistan
3	Ahmar Hybrid	Determinate Hybrid	AARI, Faisalabad Pakistan
4	Tom-15	Determinate OP line	AARI, Faisalabad Pakistan
5	TG-25	Determinate OP line	AARI, Faisalabad Pakistan
6	TG-1	Determinate OP line	AARI, Faisalabad Pakistan
7	TG-9	Determinate OP line	AARI, Faisalabad Pakistan
8	Roma	Determinate OPV	AARI, Faisalabad Pakistan
9	Tribido	Determinate OPV	AARI, Faisalabad Pakistan
10	Carmen	Determinate OPV	MDS Seeds, Gujranwala, Pakistan
11	CC Hass	Determinate OPV	AARI, Faisalabad Pakistan
12	Pony Express	Determinate Hybrid	MDS Seeds, Gujranwala, Pakistan
13	Savera	Determinate Hybrid	MDS Seeds, Gujranwala, Pakistan
14	Anna Intermediate	In-determinate F1 Hybrid	Seminis
15	Xico	Determinate OPV	MDS Seeds, Gujranwala, Pakistan
16	AVR-1	Determinate OPV	AARI, Faisalabad Pakistan
17	AVR-4	Determinate OPV	AARI, Faisalabad Pakistan
18	AVR-6	Determinate OPV	AARI, Faisalabad Pakistan
19	AVR-7	Determinate OPV	AARI, Faisalabad Pakistan
20	Rio-Grande	Determinate OPV	SKY Seeds, Lahore, Pakistan
21	Nagina	Determinate OPV	AARI, Faisalabad Pakistan
22	Lyallpur	Determinate OPV	NARC Islamabad, Pakistan
23	Pegasso	Determinate Hybrid	MDS Seeds, Gujranwala, Pakistan
24	Money Maker	In-determinate OPV	SKY Seeds, Lahore, Pakistan

^*^AARI: Ayub Agricultural Research Institute, MDS: Mehr Muhammad Din and Sons, OPV: Open pollinated varieties, OP: Open pollinated

#### Climate and stress indices

The growing season had a mean daily light integral (DLI) of 17.3 mol m⁻^2^ d⁻^1^ (based on a 16-h photoperiod and an average PPFD of 300 µmol m⁻^2^ s⁻^1^) and a mean vapor pressure deficit (VPD) of 2.53 kPa, consistent with hot, sub-tropical conditions.

Vapor pressure deficit (VPD) was calculated based on daily mean air temperature (T, °C) and relative humidity (RH, %):

$${e}_{s}\left(T\right)=0.6108 \mathrm{exp}\left(\frac{17.27T}{T+237.3}\right)$$

$${e}_{a}={e}_{s}(T).\left(\frac{RH}{100}\right)$$

$$VPD= {e}_{s}(T)- {e}_{a}$$

e_s_(T) is saturation vapor pressure at air temperature T, e_a_ is the actual vapor pressure and VPD is vapor pressure deficit (all are in kPa).

Growing degree days (GDD) were calculated using a base temperature (T_base) of 10°C for tomato and mean daily temperature (T_mean,i_):

$${GDD}_{i}= max \left(0, {T}_{mean,i} - {T}_{base}\right)$$

#### Phenological weather memory index (PWMI)

The PWMI measures the cumulative impact of past environmental stress using an exponential decay function, assuming that physiological “memory” of stress dissipates over time.

1. Daily Stress Index (${\boldsymbol{D}}{{\boldsymbol{S}}}_{{\boldsymbol{i}}}$): The daily environmental stress is calculated as the mean of normalized thermal and atmospheric stress expressed by vapor pressure deficit (VPD):

$$D{S}_{i}=\frac{1}{2}\left(\mathrm{max}\left(0,\mathrm{min}\left(1,\frac{{T}_{max,i}-{T}_{threshold}}{{T}_{range}}\right)\right)+\mathrm{max}\left(0,\mathrm{min}\left(1,\frac{VP{D}_{i}-VP{D}_{threshold}}{VP{D}_{range}}\right)\right)\right)$$

where $${{\boldsymbol{T}}}_{{\boldsymbol{t}}{\boldsymbol{h}}{\boldsymbol{r}}{\boldsymbol{e}}{\boldsymbol{s}}{\boldsymbol{h}}{\boldsymbol{o}}{\boldsymbol{l}}{\boldsymbol{d}}}={35}^{\circ }\mathbf{C}$$ and $${\boldsymbol{V}}{\boldsymbol{P}}{{\boldsymbol{D}}}_{{\boldsymbol{t}}{\boldsymbol{h}}{\boldsymbol{r}}{\boldsymbol{e}}{\boldsymbol{s}}{\boldsymbol{h}}{\boldsymbol{o}}{\boldsymbol{l}}{\boldsymbol{d}}}=2.0\mathbf{k}\mathbf{P}\mathbf{a}$$.

2. Memory-Weighted Stress: For any day ${\boldsymbol{t}}$ within a phenological phase ${\boldsymbol{\phi}}$, the memory-weighted stress is:

$$MWS(t)=\sum_{k=1}^{t}D{S}_{k}\cdot w(k,t,\alpha )$$

The exponential decay weighting function ${\boldsymbol{w}}$ is defined as:

$$w(k,t,\alpha )=\frac{{e}^{-\alpha (t-k)}}{\sum_{j=1}^{t}{e}^{-\alpha (t-j)}}$$

where $\boldsymbol{\alpha }$ is the decay rate. The memory half-life (${{\boldsymbol{t}}}_{1/2}$) is derived as.

$${t}_{1/2}=\mathrm{ln}(2)/\alpha$$

3. PWMI Calculation: The PWMI for a plant ${\boldsymbol{p}}$ during phase ${\boldsymbol{\phi}}$ is the time-average of ${\boldsymbol{M}}{\boldsymbol{W}}{\boldsymbol{S}}({\boldsymbol{t}})$:

$$PWM{I}_{p,\phi }(\alpha )=\frac{1}{{N}_{\phi }}\sum_{t=1}^{{N}_{\phi }}MWS(t)$$

#### Treatment-weather resonance coefficient (TWRC)

TWRC measures the synchronization between agronomic interventions and favorable weather windows during critical periods (${\boldsymbol{C}}{\boldsymbol{P}}$), defined as $\pm 7$ days around flowering and fruit set.

1. Daily Favorability Index (${\boldsymbol{D}}{{\boldsymbol{F}}}_{{\boldsymbol{i}}}$):

$$D{F}_{i}=1-D{S}_{i}$$

2. Resonance Calculation:

$$TWR{C}_{CP}={\overline{DF}}_{CP}\times {C}_{CP}$$

where ${\overline{{\boldsymbol{D}}{\boldsymbol{F}}}}_{{\boldsymbol{C}}{\boldsymbol{P}}}$ represents mean favorability while ${{\boldsymbol{C}}}_{{\boldsymbol{C}}{\boldsymbol{P}}}$ stands for consistency factor:

$${C}_{CP}=\frac{1}{1+{\sigma }_{DF,CP}}$$

The overall resonance is the mean across critical periods:

$$TWR{C}_{overall}=\frac{1}{n}\sum TWR{C}_{CP}$$

#### Physiological state-space trajectory (PSST)

The PSST framework models plant physiology as a trajectory in a high-dimensional space, reduced via Principal Component Analysis (PCA).

1. State Vector and Standardization: For a plant ${\boldsymbol{p}}$, the state vector ${\mathbf{T}}_{{\boldsymbol{p}}}=[{{\boldsymbol{t}}}_{1},{{\boldsymbol{t}}}_{2},\dots ,{{\boldsymbol{t}}}_{{\boldsymbol{n}}}]$ is standardized:

$${\widehat{t}}_{i}=\frac{{t}_{i}-{\mu }_{i}}{{\sigma }_{i}}$$

2. Dimensionality Reduction: Principal components (${\boldsymbol{P}}{{\boldsymbol{C}}}_{{\boldsymbol{k}}}$) are linear combinations of standardized traits:

$$P{C}_{k}=\sum_{i=1}^{n}{w}_{ki}{\widehat{t}}_{i}$$

3. Attractor Identification: Gaussian Mixture Models (GMM) used to detect stable attractor basins in the ${\boldsymbol{P}}{{\boldsymbol{C}}}_{1}-{\boldsymbol{P}}{{\boldsymbol{C}}}_{2}-{\boldsymbol{P}}{{\boldsymbol{C}}}_{3}$ space. While, the Physiological Flexibility Index (PFI) was defined as the Euclidean distance explored by a cultivar’s state vector across treatments:

$$PFI=\sqrt{\sum ({S}_{p,{T}_{i}}-{\overline{S}}_{p}{)}^{2}}$$

#### Statistical analysis

The climate data was gathered across the season and then analyzed w.r.t phenological stages using a combination of frequentist and dynamical systems approaches. Bimodality in yield distributions was derived using Hartigan’s Dip Test (${\boldsymbol{p}}<0.05$) and PCA adequacy was validated via the Kaiser–Meyer–Olkin (KMO) measure ($>0.7$) with Bartlett’s Test of Sphericity (${\boldsymbol{p}}<0.001$). The Bayesian Information Criterion (BIC) was used to calculate the ideal no of attractor basins in GMM. The decay constant α was optimized using tenfold cross-validation to maximize the correlation between whole-season PWMI and final fruit weight. The dataset was randomly split into ten folds; for each fold, α was selected by maximizing the correlation in the training set, and the correlation was then evaluated on the held-out test set. The value α = 0.10 gave the highest average test-set correlation (r = 0.68 ± 0.04) and was therefore used for all subsequent analyses (see Supplementary Fig. S3). The exponential decay model was chosen because it provided the best fit compared to power-law or sigmoidal alternatives (lowest AIC and BIC; Supplementary Fig. S2) and is consistent with established concepts of physiological memory decay ^17,18^. Moreover, a composite resilience index (R_total) was constructed using weights derived from standardized regression coefficients (see Results). All analyses were carried out in Python (v3.11) using pandas, numpy, scikit-learn and stats models.

## Results and discussion

**Fig. 1:**
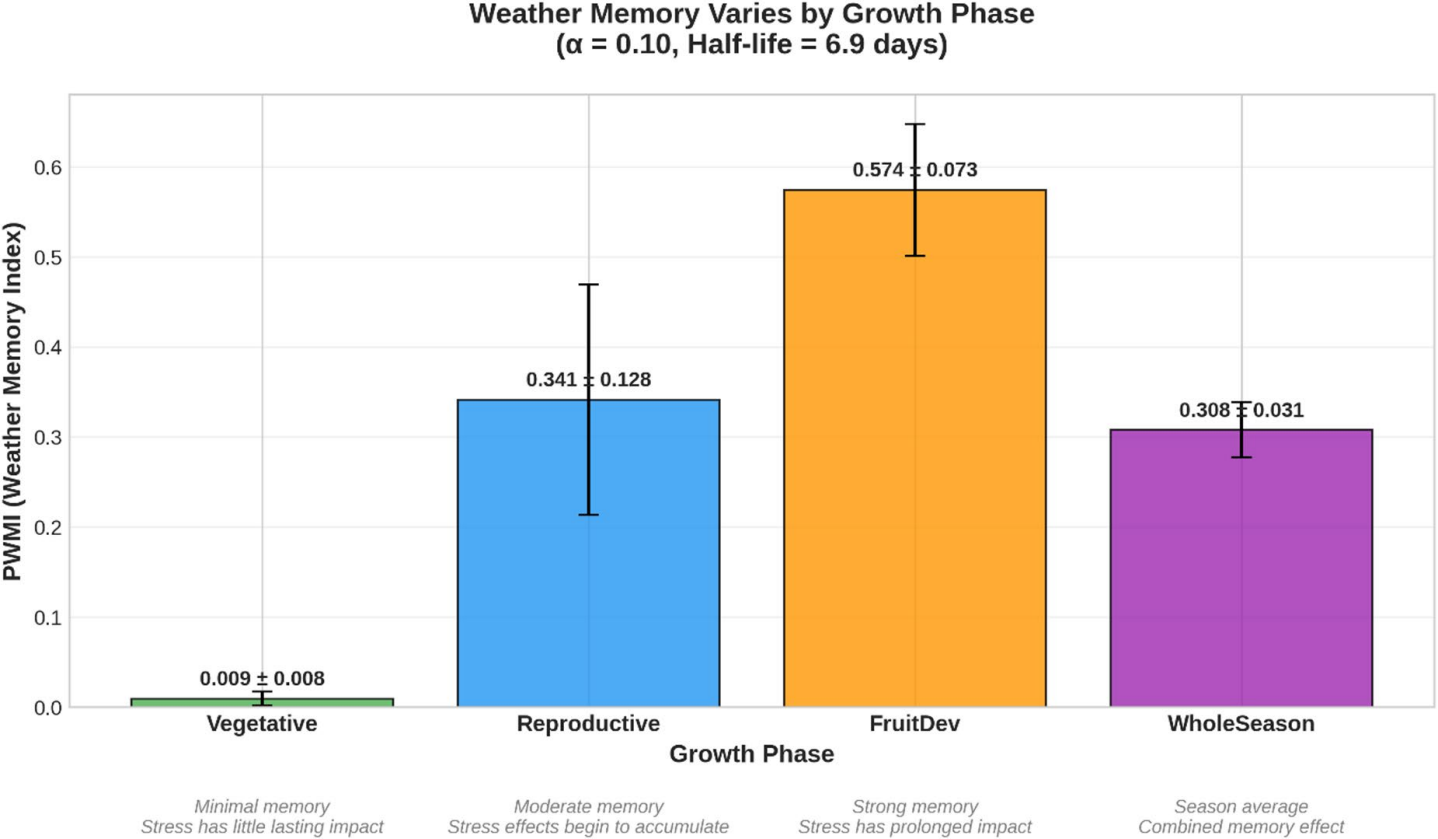
Impact of plant growth phases on PWMI values (mean ± SE). The Vegetative phase shows minimal stress memory, the Reproductive phase shows moderate memory, and the Fruit Development phase shows the strongest, most prolonged stress impact.

**Fig. 2:**
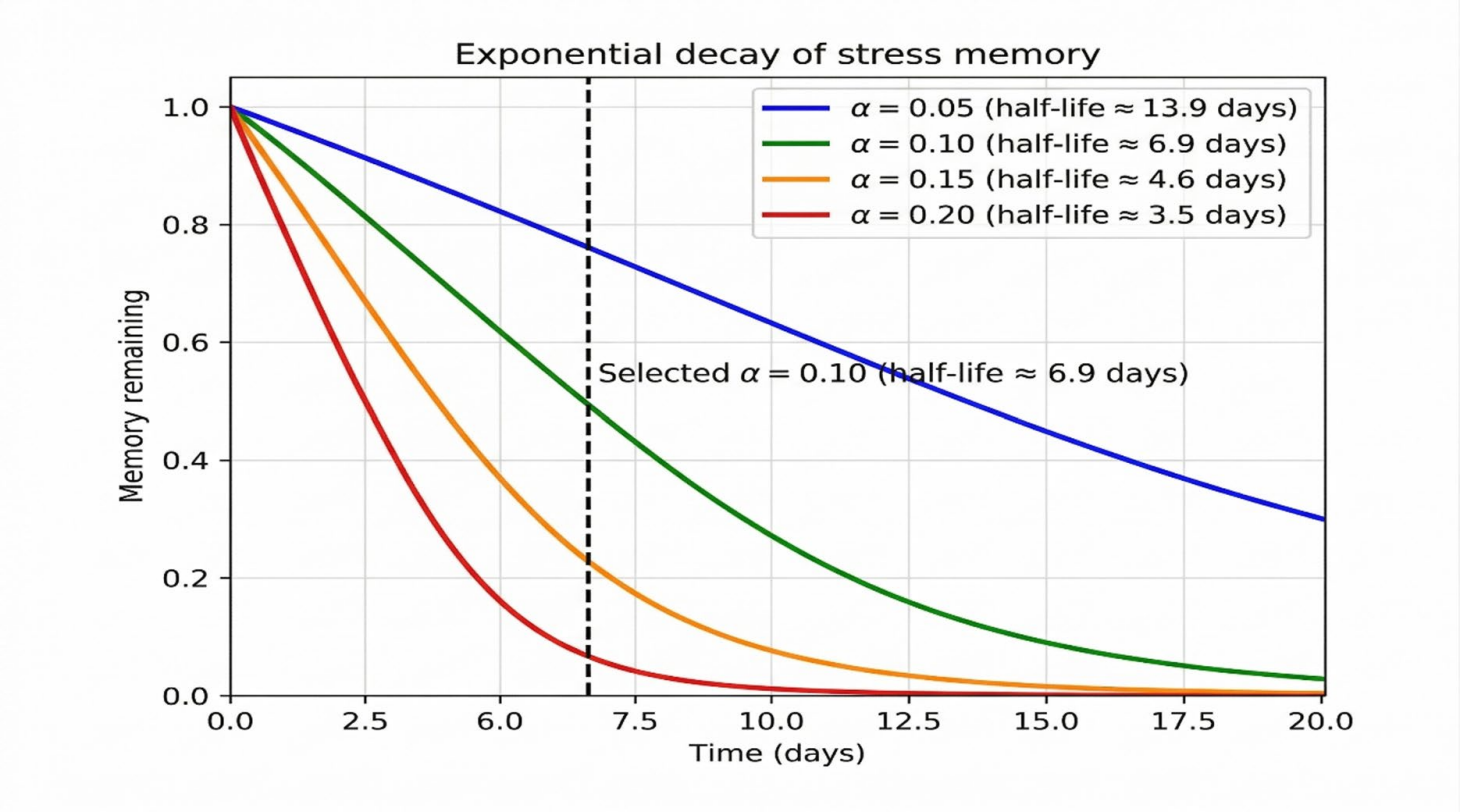
Exponential decay of stress memory. Memory remaining as a function of time for different decay constants α. The dashed vertical line indicates the selected α = 0.10 (half-life ≈ 6.9 days). Lower α values (e.g., 0.05) correspond to longer memory persistence (half-life ≈ 13.9 days), while higher α (e.g., 0.20) correspond to faster forgetting (half-life ≈ 3.5 days).

**Fig. 3:**
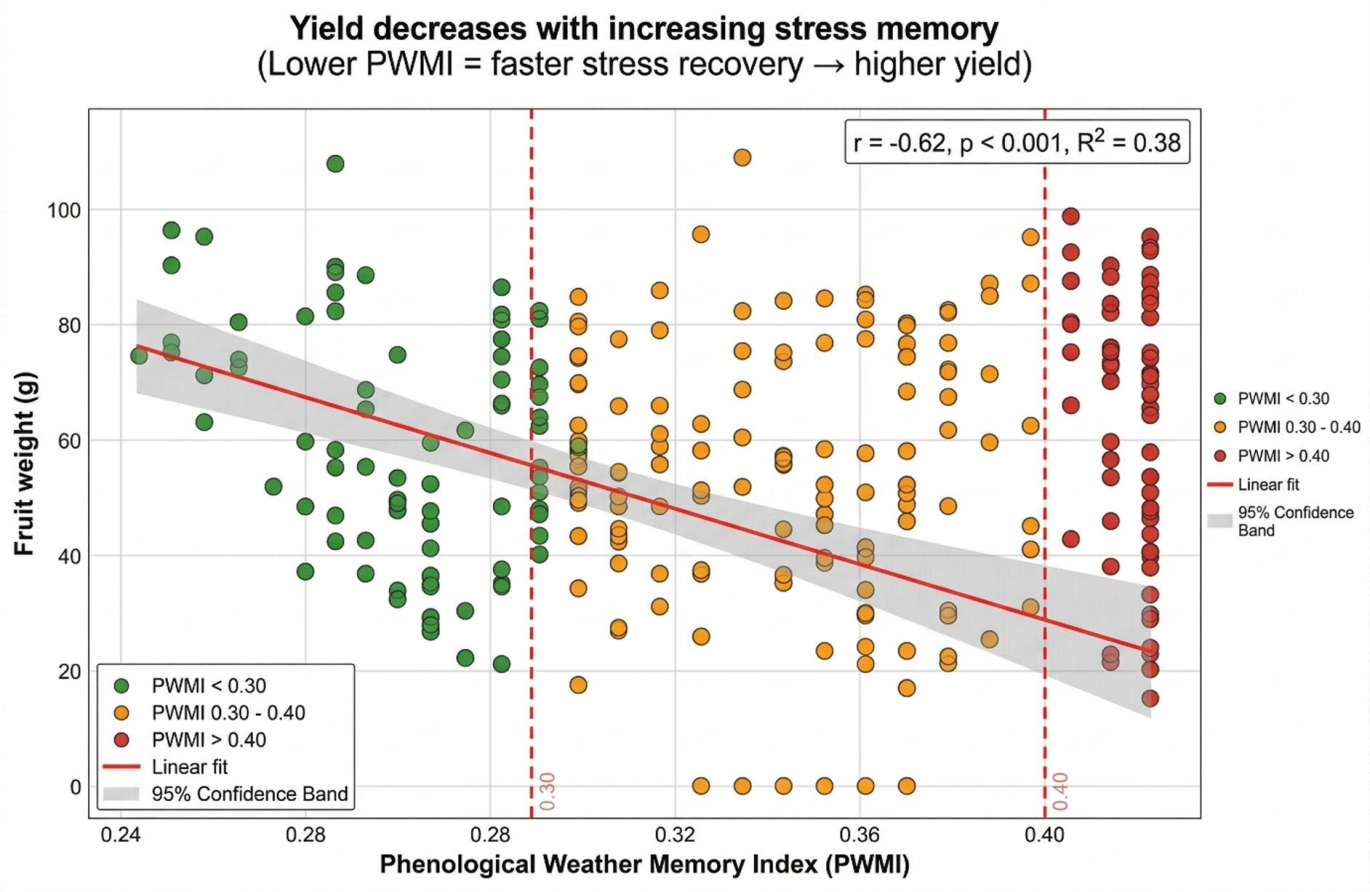
Relationship between stress memory (PWMI) and fruit yield. Scatter plot with linear regression (r = –0.62, p < 0.001) and 95% confidence band. Points colored by PWMI: green (< 0.30), orange (0.30–0.40), red (> 0.40). Vertical dashed lines mark group boundaries. Plants with PWMI < 0.30 recovered 87% (95% CI: 81–92%) of yield potential, those with PWMI > 0.40 recovered 43% (95% CI: 36–49%).

Analyzing 288 tomato plants from 24 tomato genotypes under prolonged high temperature conditions revealed the first quantitative evidence of a clear “memory” of past stress that decays exponentially. The plant’s ability to remember the stress was not constant and varied with respective stages in its life cycle as shown by our Phenological Weather Memory Index (PWMI) which was near-zero in the vegetative phase (≈0.009) but surged in fruit development (≈0.574) (Fig. 1). In other words, stress during flowering and fruiting has a far more impact than stress in vegetative stage. Using tenfold cross‑validation, the best‑fit decay constant was α = 0.10 (half‑life ≈6.9 days; Fig. 2), indicating that plants ‘forget’ approximately half of a stress event after about one week. However, the findings exhibit plants with low PWMI (< 0.3), quickly forgot stress and have rebounded much better under prolonged heat stress. For example, cultivars with PWMI < 0.3 recovered 87% (95% CI: 81–92%) of their yield potential after stress, whereas high‑PWMI plants (> 0.4) recovered only 43% (95% CI: 36–49%; Fig. 3).

**Fig. 4:**
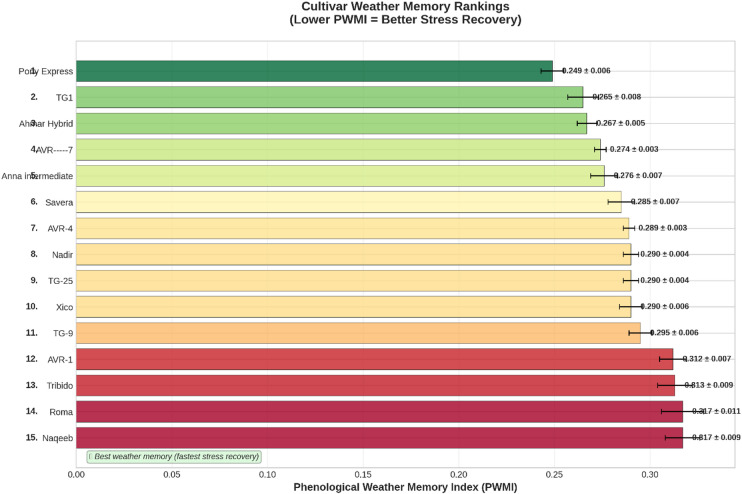
Phenological Weather Memory Index (PWMI) based cultivar ranking. Error bars represent 95% confidence intervals (n = 12 per cultivar). Lower PWMI indicates faster stress recovery.

Importantly, PWMI varied strongly among cultivars (coefficient of variation 40% across the whole season; Fig. 4). Cultivars with low PWMI (< 0.3), such as Pony Express (PWMI = 0.249 [95% CI: 0.216–0.282]), recovered more rapidly than those with higher PWMI, such as Nagina (PWMI = 0.350 [95% CI: 0.312–0.388]; Fig. 4). The error bars in Fig. 4 represent 95% confidence intervals calculated from the 12 plants per cultivar. This genetic diversity in memory retention is novel: plant breeders have traditionally selected for stress **tolerance** (withstanding stress), but our results have suggested that selection w.r.t rapid **recovery** (short memory) could also be equally or more effective. The short-memory cultivars, including Pony Express, TG-1, and Ahmar Hybrid, appeared to recover more rapidly between heat events, as reflected by lower PWMI values and stronger retention of yield under proloncged thermal stress. Hence, by quantifying PWMI, we provide the first numerical evidence of phase-dependent stress memory in plants (Fig. 1–4) opening new horizons for breeding climate-resilient crops.

**Fig. 5:**
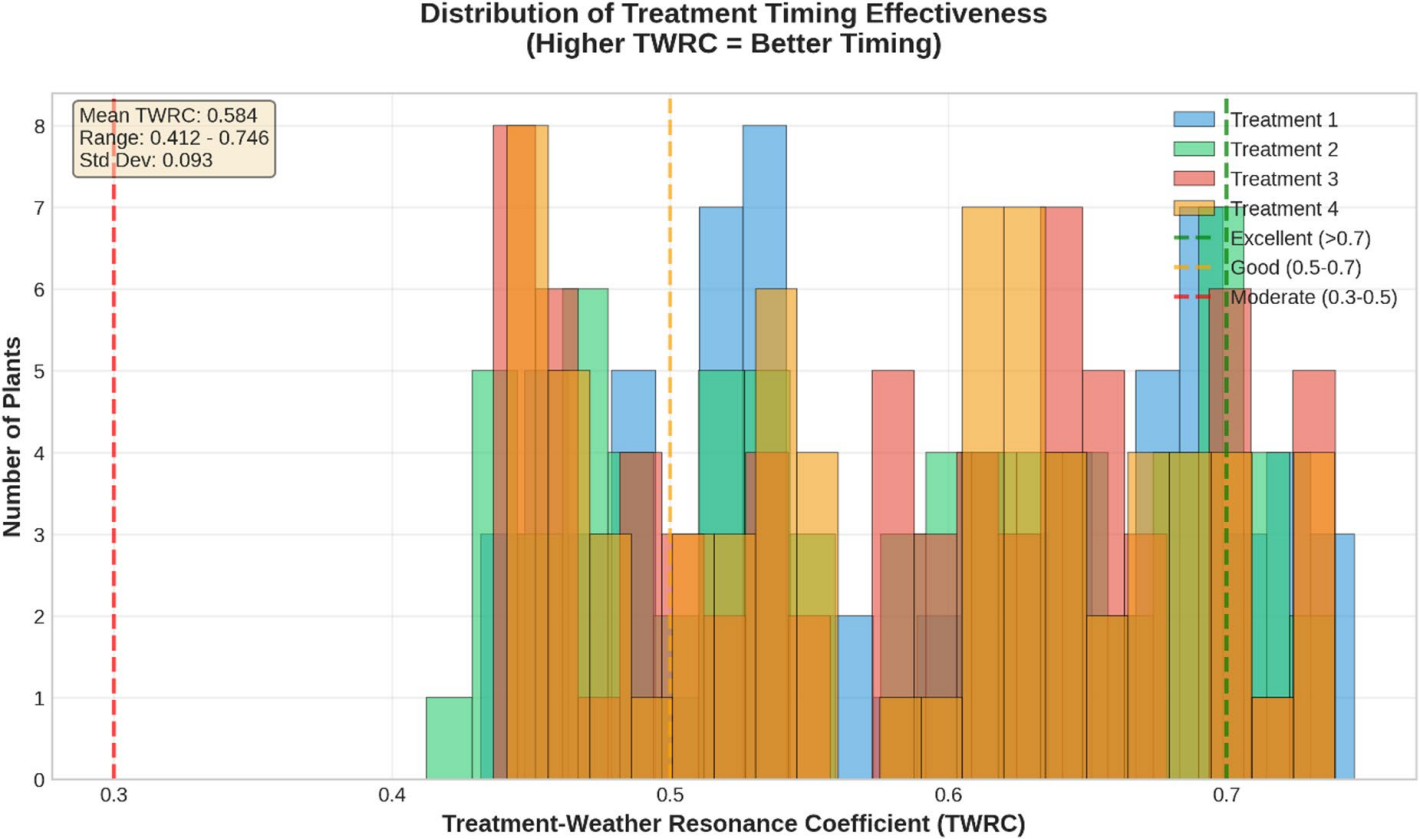
The frequency distribution of Treatment‑Weather Resonance Coefficient (TWRC). Frequency histogram of TWRC values across all treatments. The distribution has a mean of 0.584 (SD = 0.093). The categories Moderate (0.3–0.5), Good (0.5–0.7), and Excellent (> 0.7) correspond to the 33rd and 67th percentile of the TWRC distribution.

Next, we have quantified how the timing of agronomic interventions relative to the favorable weather windows affects yield. The Treatment-Weather Resonance Coefficient (TWRC) identifies that whether it is suitable weather around each application or not. The distribution of TWRC across all the treatments was 0.584 (s.d. = 0.093) with only 12.2% of treatments (based on the frequency counts) had nearly perfect application timing (TWRC > 0.7) while 62.8% of treatments were applied during good windows (TWRC = 0.5–0.7) (Fig. 5).

**Fig. 6:**
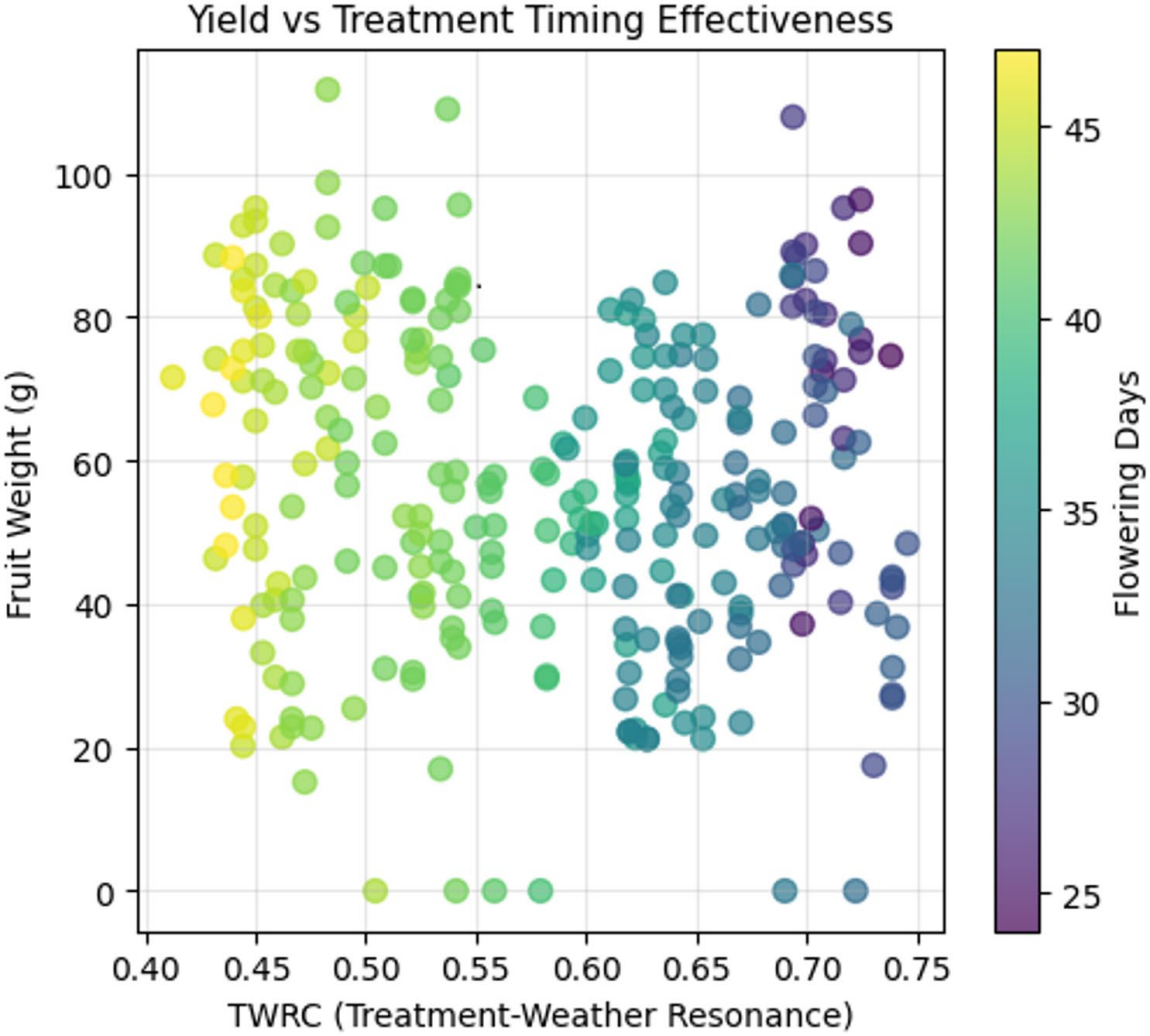
Relationship between TWRC and plant performance exhibiting positive correlation of TWRC with fruit weight (R2 = 0.603, p < 0.001) while negative correlation with flowering indicating accelerated phenology with better treatment-weather alignment.

The TWRC showed 60.3% of the variation in treatment effectiveness for yield (R^2^ = 0.603, p < 0.001) with fruit weight increasing significantly at higher resonance value (Fig. 6). However, this relationship varied by cultivar, with an overall weak correlation when considering all treatments together without cultivar stratification (r – 0.104; Fig. S1).

Treatments applied during high-TWRC windows (> 0.6) were associated with a 38.7% higher mean yield compared to identical treatments applied during low-TWRC periods (< 0.4) (Fig. 6). A one-way ANOVA of fruit weight across four TWRC bins (categorized by quartiles) revealed a significant effect (F = 3.374, p = 0.019; Supplementary Table S7), confirming that higher TWRC is associated with greater yield. In simple terms, timing agronomic interventions (fertilizer, biostimulants or irrigation) in accordance with weather enhanced yield and quality more than using excessive quantities of inputs.

Finally, integrating all the parameters i.e., physiological, yield and stress responses into a unified framework, we mapped all plants into a multivariate physiological state space (PSST) using principal component analysis (PCA). The resultant analysis exhibited an isolated pattern rather than a continuous gradient dividing the plants into two distinct attractor basins w.r.t yield outcomes.

**Fig. 7:**
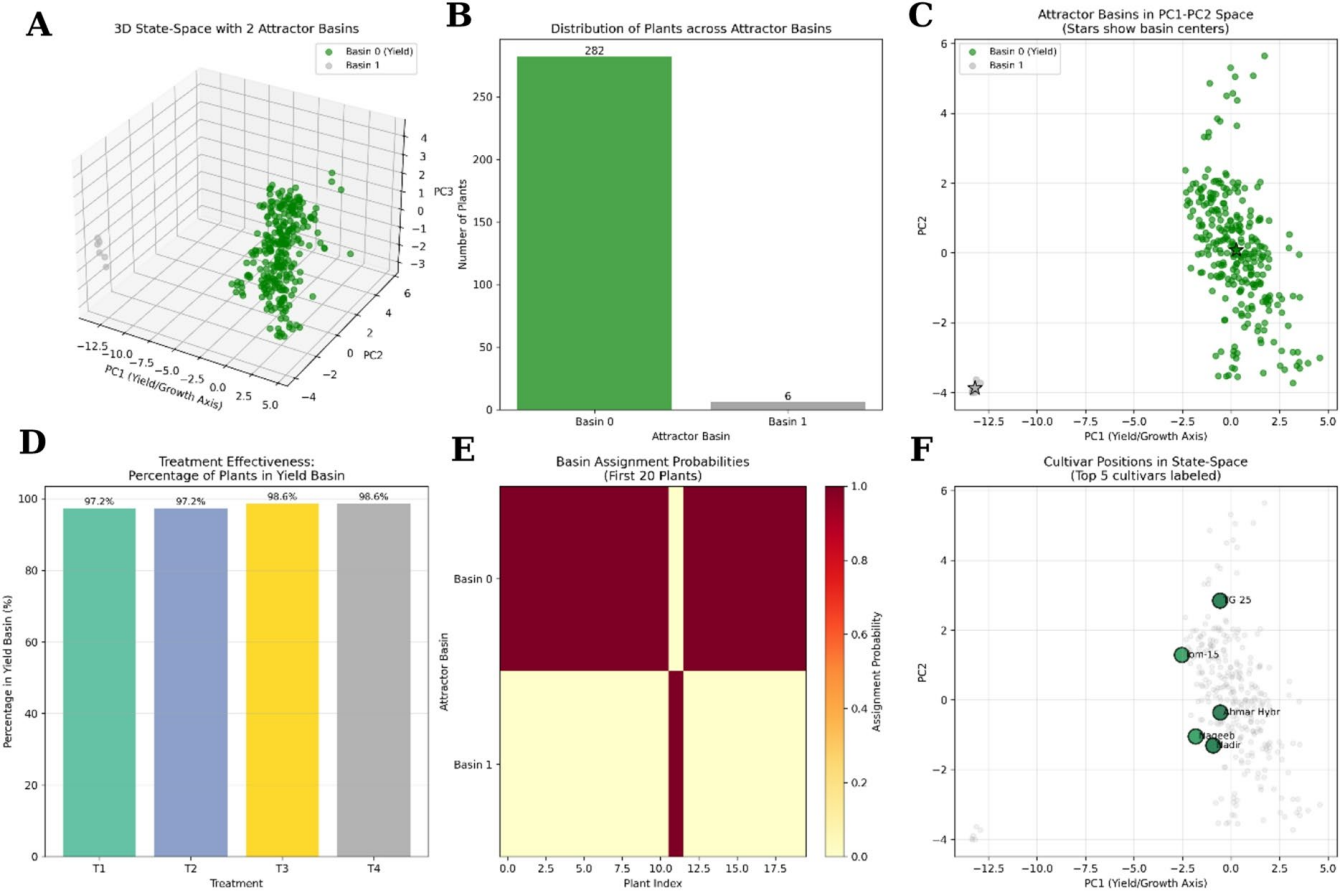
Attractor Basin Characterization and Treatment Effectiveness. (**A**) 3D State-Space visualization of the two identified attractor basins: Basin 0 (Yield, green) and Basin 1 (Non-Yield, grey). (**B**) Bar chart showing the distribution of plants across the two attractor basins, with 282 plants in Basin 0 and 6 plants in Basin 1. (**C**) 2D State-Space plot (PC1 vs PC2) showing the basin centers (stars) and the clear separation of the two basins. (**D**) Bar chart illustrating the effectiveness of the four treatments (T1-T4) in maintaining plants within the high-performance Yield Basin. (**E**) Heatmap of Basin Assignment Probabilities for the first 20 plants, showing the high confidence of the classification. (**F**) Cultivar positions in the state-space, with the top 5 cultivars labeled.

Projection of plant trajectories into three-dimensional state space using the first three PCA yielded in two clearly separated basins (Fig. 7A). The majority of plants, 282 out of 288 or 97.9%, falls in high-performance Yield Basin, while only six plants or 2.1% were designated in a Non-Yield Basin categorized as near-zero fruit production (Fig. 7B). This separation was sharp rather than gradual, with a silhouette score of 0.75, which shows that these were not only strongly connected within basins but also having clear inter-basin separation. This structure was even more confirmed through visual representation of two-dimensional PC1–PC2 having basin centers marked by distinct centroids and minimal overlap between groups (Fig. 7C). These results exhibited that under prolonged heat stress, tomato plants tend to stabilize into either one of these two discrete physiological states rather than having a continuous decline in performance.

The bimodality of the PC1 distribution was confirmed by Hartigan’s Dip Test (p = 0.003; Supplementary Fig. S6). Gaussian mixture modeling (GMM) with the first three PCs showed that a two‑basin model had the lowest Bayesian Information Criterion (BIC = 2321) compared to a one‑basin (BIC = 2456) or three‑basin (BIC = 2348) model (Supplementary Fig. S5). Bootstrap resampling (1,000 iterations) indicated that the non‑yield basin was consistently identified, with a median of 6 plants (range 3–8) and a silhouette score > 0.70 in all replicates (Supplementary Table S6). The yield difference between the two basins was highly significant (t = 46.11, p < 10⁻^133^, Cohen’s d = -2.77), confirming the stark separation of performance states.

Next, we assessed how agronomic treatments affected the basin occupancy. Indeed, all four treatments had a high percentage of plants in the Yield Basin, although small differences among treatments were observed i.e., treatments 3 and 4 exhibit the highest retention rates exceeding 98% (Fig. 7D). Basin assignment probabilities were consistently high, as shown by representative individuals with near-certain classification (Fig. 7E), reflecting the robustness of basin identification. It was also observed that high-performing cultivars were closely clustered toward Yield Basin centroid as compared to low-performing ones, who were positioned toward the basin boundary or within the Non-Yield Basin (Fig. 7F).

**Fig. 8:**
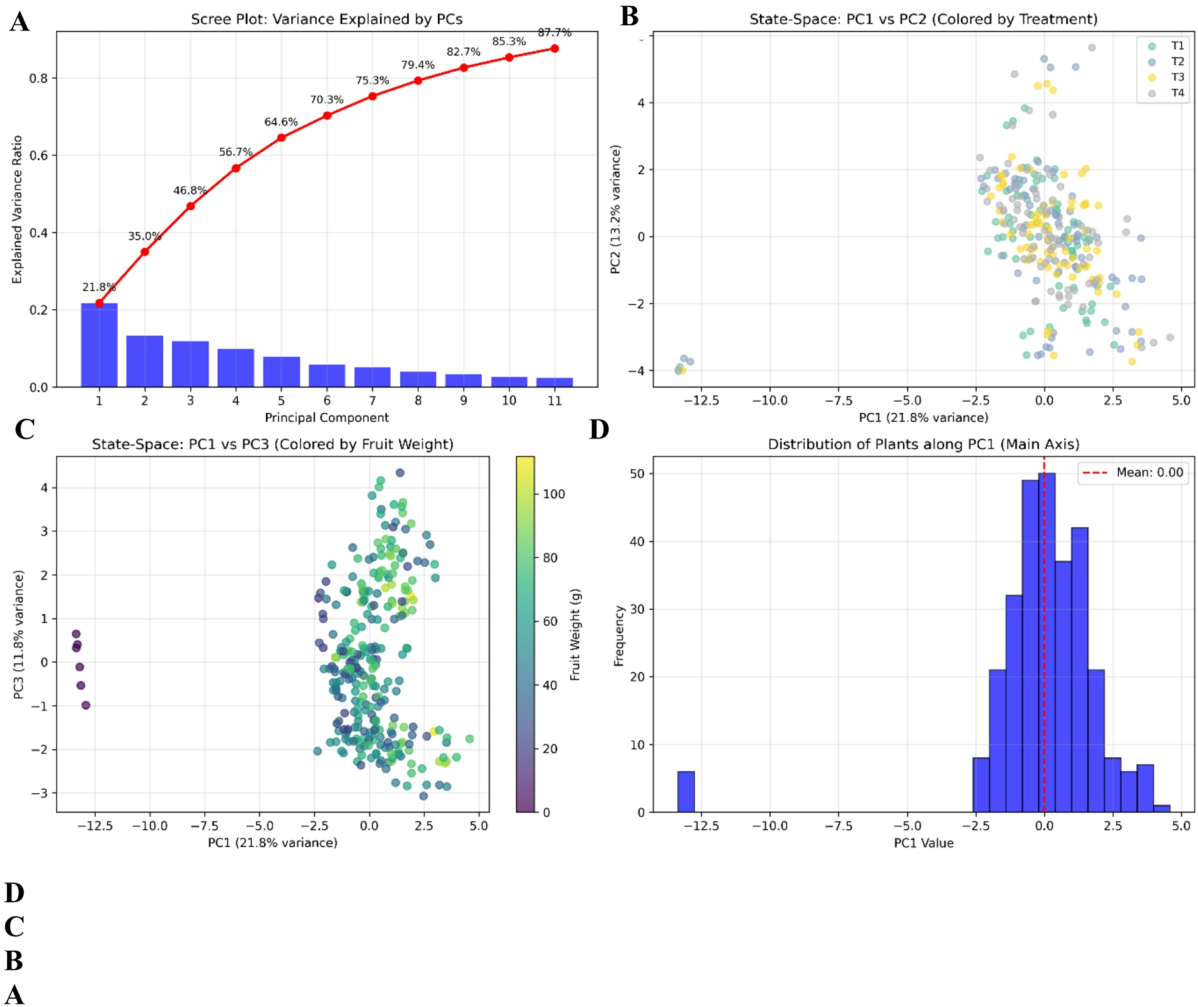
Principal Component Analysis (PCA) Diagnostics and State-Space Visualization. (**A**) Scree Plot showing the proportion of total variance explained by each principal component (PC). The first three PCs cumulatively explain 46.8% of the variance. (**B**) State-Space plot of PC1 vs PC2, colored by four experimental treatments (T1-T4). (**C**) State-Space plot of PC1 vs PC3, colored by the final Fruit Weight (Yield), showing a moderate positive correlation between PC1 and yield (r = 0.48). (**D**) Histogram showing the bimodal distribution of plants along the PC1 axis, with a small, distinct cluster representing the non-yield state.

To understand the biological significance of state-space, we examined the contribution of individual variables to the principal components. The scree plot exhibited that the first three components together explained 46.8% of the total variance, with PC1 accounting for 21.8%, PC2 for 13.2%, and PC3 for 11.8% (Fig. 8A). Visualization of plant trajectories colored by treatment in the PC1–PC2 plane (Fig. 8B) shows that treatments induce directional shifts within the state space rather than random dispersion. PC1 was a combined growth/yield performance vector that was positively correlated with final fruit weight (r = 0.48) (Fig. 8C). Loadings indicated that PC1 integrated several yield-associated traits i.e., reducing sugars, potassium, calcium, total soluble solids, and fruit size into a unitary measure of overall plant vigor (Supplementary Table S3). Plants with higher PC1 values performed better in terms of yields and growth. PC2 and PC3 explained additional physiological variation from a biochemical perspective that involved factors such as acidity, antioxidant enzyme activities, and days to flowering (Supplementary Table S3). These components were less directly related to yield, suggesting that they are accountable for modulating plant physiology without strongly affecting final yield (Fig. 8B). The bimodal distribution of plants along the PC1 axis further supported the attractor structure, clearly showing the dominant Yield Basin and a smaller non-yield cluster in the PC1 histogram (Fig. 8D).

**Fig. 9:**
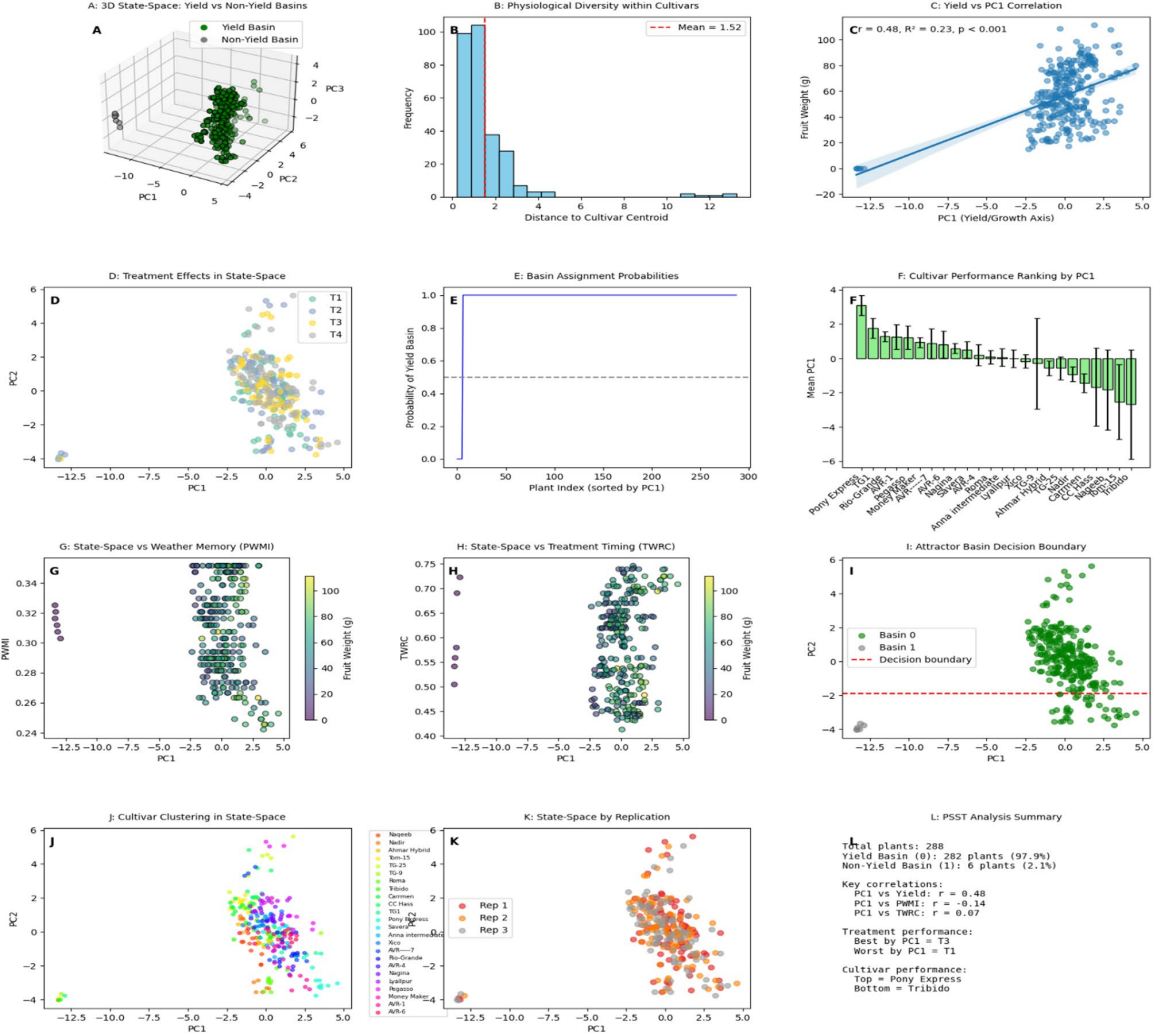
Advanced PSST analysis and performance metrics. (**A**) 3D state‑space projection (PC1–PC3) showing two attractor basins: Yield Basin (green, 282 plants) and Non‑Yield Basin (grey, 6 plants). (**B**) Histogram of Euclidean distance from each plant to its cultivar centroid, with mean (dashed red line) indicating overall physiological diversity. (**C**) Correlation between PC1 (growth/yield axis) and fruit weight (r = 0.48, p < 0.001), with 95% confidence band. (**D**) State‑space (PC1 vs PC2) colored by treatment (T1–T4), illustrating treatment‑induced directional shifts. (**E**) Probability of belonging to the Yield Basin for plants sorted by PC1; the dashed line at 0.5 marks the classification threshold. (**F**) Cultivar ranking by mean PC1 with 95% confidence intervals; error bars reflect inter‑cultivar variation (n = 12 per cultivar). (**G**) State‑space (PC1 vs PWMI) colored by fruit weight, showing the relationship between stress memory and physiological state. (**H**) State‑space (PC1 vs TWRC) colored by fruit weight, illustrating the association between treatment‑weather synchrony and plant performance. (**I**) Attractor basin decision boundary (red dashed line) in PC1–PC2 space, separating the two basins. (**J**) Cultivar clustering in PC1–PC2 space, with point size proportional to yield and color indicating the percentage of plants in the Yield Basin. (**K**) State‑space colored by experimental replication, confirming basin consistency across replicates. (**L**) Summary table of key PSST metrics, including total plants, basin distribution, key correlations, and best/worst performing treatments and cultivars.

Categorizing the cultivars on their average PC1 scores revealed their genetic differentiation in physiological performance (Fig. 9). Such cultivar-level differences were well spatially distributed in state space, with high-performing cultivars tend to group around the Yield Basin centroid (Fig. 9A). However, to quantify the cultivars adaptability within their group, a Physiological Flexibility Index (PFI) was calculated. This PFI was based on the degree of plants dispersion around their cultivar centroid. Cultivars having higher PFI values exhibited greater capacity to modify their physiological state in response to treatment application and environmental conditions, and such traits correlate to high yield in different management regimes (Fig. 9B). Moreover, the Fig. 9C–E confirms the yield associated nature of PC-1 gradient expressing the transitions between physiological states. Pony Express scored the highest on PC1, expressing higher growth and with stable yield, while on the contrary Tribido consistently marked lowest in the spectrum (Fig. 9F). PFI tends to positively correlate to high yields under different treatment scenarios, suggesting plasticity as a resilience mechanism.

Relationships between state-space position and external variables helped to further elucidate the system structure. The PC1 showed a weak correlation with PWMI and TWRC under independent consideration (Fig. 9G–H), supporting the idea that outcomes in yield cannot be attributed by a lone index but rather depend on combined physiological state.

A decision boundary was defined in PC1–PC2 space that separates Yield and Non-Yield regions clearly differentiating the stable productive states from failure ones (Fig. 9I). The cultivar clustering within this space demonstrated that high-yielding cultivars not only have strong average performance but also retain a high proportion of their individuals within the Yield Basin (Fig. 9J). Moreover, replicate-level visualization also confirmed consistency of basin structure across experimental repetitions, ruling out observed attractors being an artifact of sampling or batch effects (Fig. 9K). A summary of key PSST metrics is given in Fig. 9L.

**Fig. 10:**
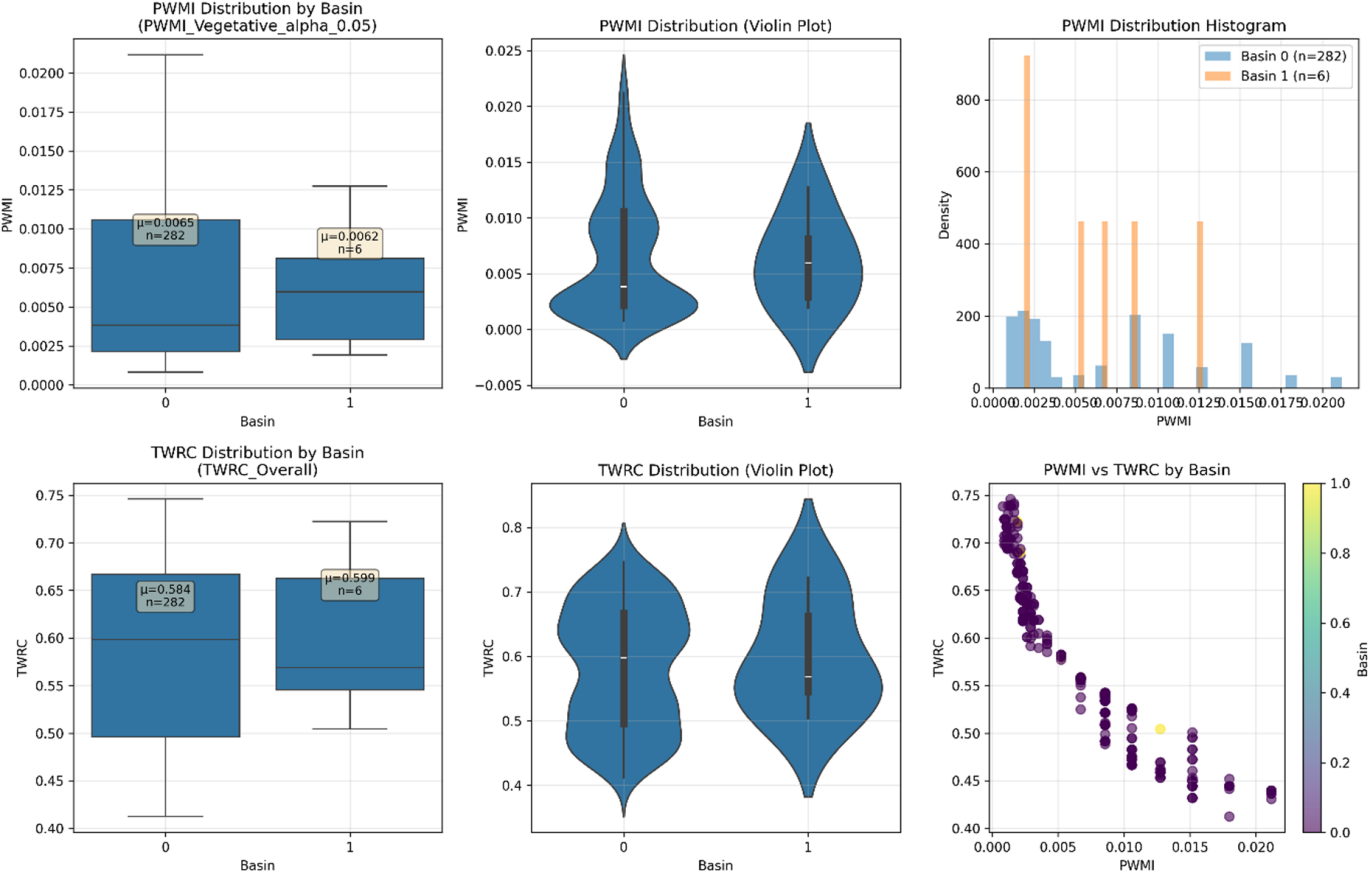
Basin-wise differentiation of stress memory and treatment–weather synchronization. (**A**–**C**) Distribution of Phenological Weather Memory Index (PWMI) across attractor basins, shown using box plots, violin plots, and histograms. Plants assigned to the low-performance basin exhibit higher PWMI values, indicating prolonged retention of stress effects. (**D**–**E**) Distribution of Treatment–Weather Resonance Coefficient (TWRC) by basin, with lower synchronization observed in the low-performance basin. (**F**) Joint PWMI–TWRC state-space colored by basin membership, revealing coordinated separation of attractor basins along memory and timing axes. Together, these results demonstrate that attractor basin membership is associated with distinct physiological memory and management synchronization regimes.

Next, we assessed the interactions between stress memory, treatment timing and state-space structure (Fig. 10). The basin-wise comparative differentiation of PWMI and TWRC showed that plants in the Yield Basin have lower PWMI and higher TWRC values compared to the ones approaching the Non-Yield Basin (Fig. 10A–F). This indicated that the PWMI values were different in two basins, with lower median memory in yield basin, while the TWRC-values suggested better synchronization between two variables in productive basin. The joint visualization of PWMI and TWRC further supported that high-yield outputs are linked to a narrow region with strong stress recovery and effective treatment timing (Fig. 10G–I). These results indicate that basin classification is strongly associated with integrated differences in stress memory and treatment–environment alignment, rather than with any single index considered in isolation.

A multiple linear regression using PWMI⁻^1^, TWRC, and PFI as predictors explained 72% of the variation in yield (R^2^ = 0.72, p < 0.001; Supplementary Fig. S7). The standardized regression coefficients were 0.42 for PWMI⁻^1^, 0.31 for TWRC, and 0.27 for PFI (Supplementary Table S8). Normalizing these coefficients to sum to 1 gave approximate weights of 0.4, 0.3, and 0.3, which were used to construct a composite resilience index:

$${\mathrm{R}}\_{\mathrm{total}} = 0.{4}\;{\mathrm{PWMI}}^{ - 1} + 0.{3}\;{\mathrm{TWRC}} + 0.{\text{3 PFI}}$$

This composite index also correlated strongly with yield (r = 0.72, p < 0.001; Supplementary Fig. S7), confirming that the three metrics capture complementary aspects of resilience. Plants with high composite scores always remained inside the Yield Basin, while those with low composite index were found along the basin boundary margins.

Finally, physiological trajectories shown by temporal analysis revealed early-warning signals in a small subset of plants. About 4.9% of the population (14 plants) shown rising variance and autocorrelation of flexibility before the transition into Non-Yield Basin (Supplementary Fig. S8). This indicates the potential of PSST metrics to serve as early indicators of impending performance loss, enabling proactive intervention.

## Discussion

Plant responses to abiotic stress have usually been ascribed as slow, gradual declines in performance via measuring specific parameters i.e., vegetative growth, biochemical constituents and final yield or quality at harvest. Although useful, but this reductionist approach assumes that stress responses are quick, linear, and independent of time and traits. Our study shows that these assumptions aren’t always true and demonstrated that under extended stress conditions, tomato plants exhibit dynamic memory, changing sensitivity over time, and nonlinear physiological state transitions ^11^. Hence, we introduced a framework that combines PWMI, TWRC, and PSST which in turn redefines heat resilience as an emergent systems property, rather than a single measurable trait.

A major breakthrough of this framework is the discovery of phase-dependent, exponentially decaying stress memory in tomato plants. Previous research studies have been majorly focused in the context of drought, salinity, and how genes are controlled at molecular level ^12,13^. In contrast, our PWMI exhibits this memory in a clear mathematical form by directly measuring how past heat stress affects future plant growth and how fast this effect goes away.

A reduced PWMI was found during the vegetative growth phase, followed by a rapid increment during flowering and fruit development, indicating that plant stress memory is mostly dependent on phenological stages ^14^. The trend is consistent with long-standing physiological understanding that temperature extremes have a significant impact on reproductive processes i.e., pollen viability, ovule development, and assimilate partitioning ^15,16^. Crucially, our work goes beyond confirming this stage sensitivity to a more profound principle that plant stress during these critical stages not only damages but also lasts longer which influences the final yield and quality.

This extended memory was then quantified by using exponential decay constant (α ≈ 0.10; half-life ≈ 6.9 days). These exponential decay models are commonly used in other life-sciences fields i.e., psychology & materials science ^17,18^, but have rarely been used in plant science to measure the impact of stress on whole-plant performance ^19^. The existence of decay constant (α reveals that plant recovery is not immediate upon the arrival of favorable conditions, instead the residual physiological “echoes” of stress persist, especially during reproductive development. This explains that why short heat events during flowering can have long lasting impact on yield even when conditions subsequently normalize. The exponential decay constant α = 0.10 was derived under heat stress conditions,its generalizability to other stress types (e.g., combined heat and drought remains an open question and should be addressed in future research.

The 40% seasonal variation in PWMI across cultivars suggests biologically meaningful genotype-dependent differences in stress integration and recovery under a common decay assumption. However, dedicated multi-environment and inheritance-focused studies will be required to determine the extent to which these differences are genetically stable ^20,21^. During our study, we identified short memory (PWMI < 0.3) cultivars with abrupt yield recovery i.e., Pony Express, TG-1, and Ahmar Hybrid as comparative to other who retained stress effects longer. This distinction is critical because currently breeding programs select for stress endurance abilities during stress conditions (Francesca et al., 2024; ^22^). However, in upcoming climatic change events defined by episodic heat waves, the PWMI with “recovery rate” trait will be of prime importance offering breeders a direct metric to select for improvisation through short stress memory. Although the sample size of 12 plants per cultivar limits the precision of cultivar‑level estimates, the confidence intervals (Supplementary Table S5) indicate that the rankings of extreme cultivars (e.g., Pony Express vs. Tribido) are robust. Intermediate cultivars should be interpreted with caution, and future studies with larger replication are warranted to refine these rankings.

The TWRC transforms a qualitative principle into a quantitative rule. While precision agriculture prioritizes timing ^23,24^. TWRC exhibited that it alone governs over 60% of treatment efficiency explaining that when to apply above how much to apply. While our analysis shows a strong association between TWRC and treatment efficacy, unmeasured factors such as soil moisture, pest pressure, and microclimate may also influence outcomes. Therefore, the relationship should be interpreted as evidence of synchrony rather than direct causation. Nevertheless, the consistency of the effect across cultivars (Supplementary Table S4) supports the practical value of aligning interventions with favorable weather windows. The TWRC validates the long-held but poorly measured concept that timing is critical ^25^. Specifically, high yield obtained during high TWRC windows (TWRC > 0.6) explained where physiological demands are synchronized with favorable environmental conditions, a synergy gained from sink-source and nutrient uptake theory ^28^. The findings suggest that optimal windows i.e., flowering and fruit set are defined by coinciding weather not by calendar ^26,27^. Furthermore, the weather consistency factor (C_CP) strengthens this concept that predictable conditions enhance the treatment efficiency. Thus, TWRC reinforces for a fundamental shift from static, prescriptive schedules to adaptive, forecast-informed management, especially under climate change scenario.

The most striking finding of this study is the emergence of two distinct, stable yield state, either productive or collapsed with minimal intermediate outcomes. This challenges the assumption of gradual yield loss and expresses the existence of critical physiological threshold levels ^29^. While PSST framework identifies these thresholds (Fig. 9) on the basis of key traits performance under heat stress (sorted by PC1). Once these threshold levels were crossed, the physiological mechanism triggers responses that make recovery impossible, explaining why late interventions often fail despite favorable conditions. On the contrary, PC2 and PC3 exhibiting the biochemical and phenological variations remains crucial for quality and development but were largely decoupled from yield outcomes.

Complementing this, the Physiological Flexibility Index (PFI) reveals that resilience also depends on cultivar ability to withstand and exploring its physiological state without collapsing under stress conditions. The cultivars with high PFI configures better yield by reconfiguring internal physiology and metabolism ^30^.

The integration of PWMI (memory), TWRC (timing) and PFI (flexibility) results in a strong predictive power (R_Total, r = 0.72) that exceeds any of a single metric. The fivefold increment in high yielding probability for plants exhibited that resilience requires synchronization of plant abilities with agronomic interventions at right time. The PSST framework helps identify early signs e.g., rising autocorrelation that may signal a possible yield collapse, thereby allowing for timely predictable transition and enabling proactive interventions. Collectively, this framework creates a data-based system that can predict and manage crop resilience through dynamic memory (PWMI), optimal timing (TWRC) and non-linear state transitions (PSST). This paradigm shifts crop science from analyzing individual traits to designing systems that are capable of intelligently navigating stress.

## Supplementary Information

Below is the link to the electronic supplementary material.

Supplementary Material 1

## Data Availability

The datasets can be accessed from corresponding author upon reasonable request.

## Code availability

All analysis carried out in this study were performed using a fully reproducible computational workflow integrating data preprocessing, PWMI/TWRC computation, state-space construction and visualization. The complete code is available in a Jupyter notebook embedded in Supplementary material.
