# Supplementary material for: Quantifying the dynamic recovery of plants through stress memory and physiological attractors
Source: Sci Rep. 2026 Apr 30;16:20061. doi: 10.1038/s41598-026-49352-y (PMC13324007; doi:10.1038/s41598-026-49352-y)
Supplement: Supplementary file 1 — Supplementary Material 1 [file 41598_2026_49352_MOESM1_ESM.docx]

**
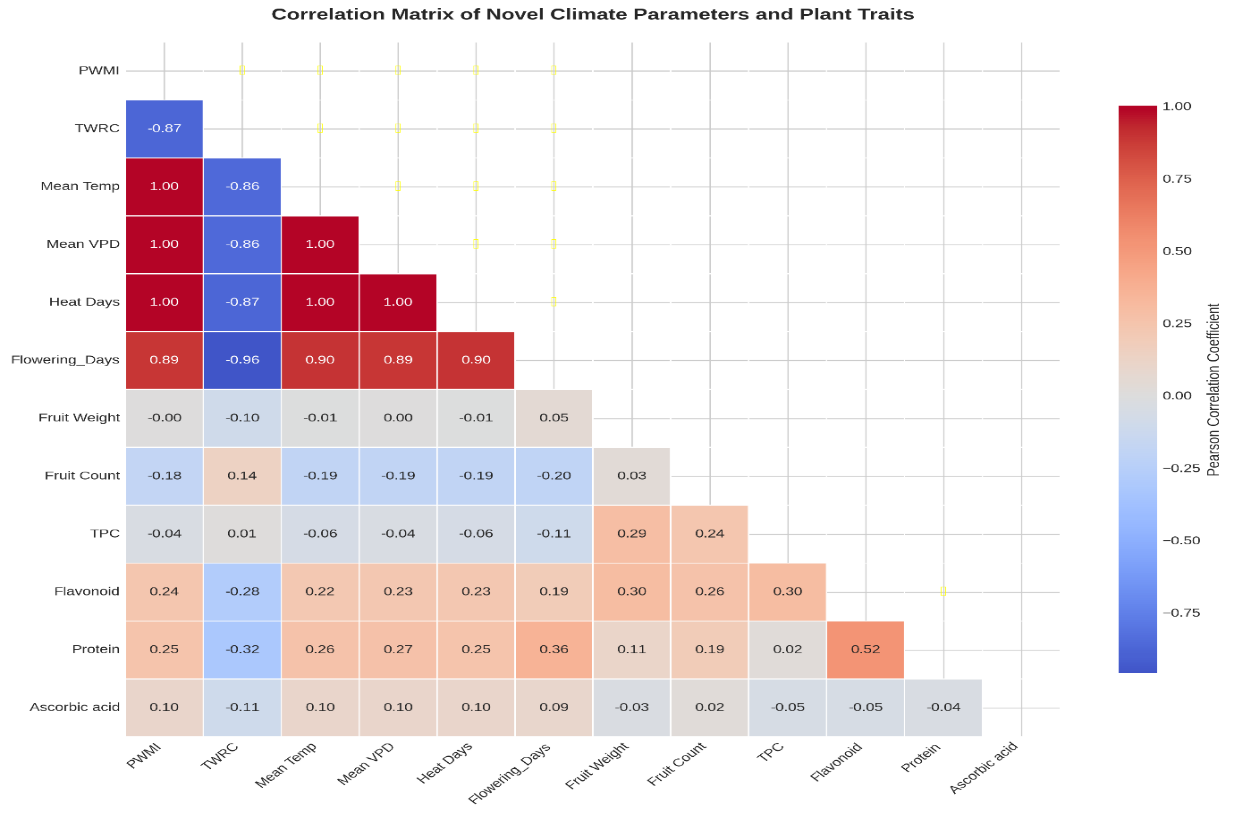
**

**Fig. S1. Correlation matrix of climate-derived indices, phenology, and plant traits.**
Heatmap showing Pearson correlation coefficients among PWMI, TWRC, seasonal climate variables, flowering time, and key yield and biochemical traits. Color scale indicates correlation strength and direction. Strong associations among climate exposure variables contrast with weaker direct correlations between indices and yield traits.


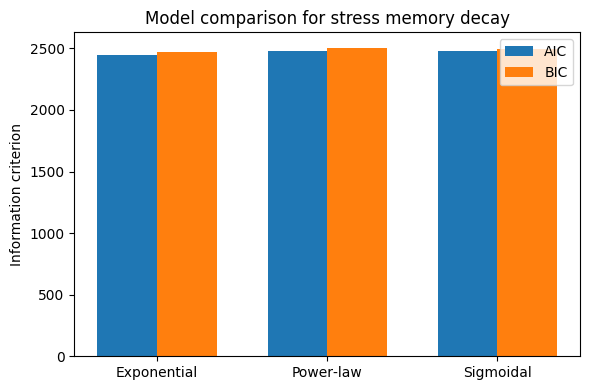


**Fig. S2. Comparison of decay models for stress memory.** Bar chart of Akaike Information Criterion (AIC) and Bayesian Information Criterion (BIC) for exponential, power‑law, and sigmoidal decay models fitted to the whole‑season stress‑memory relationship. Exponential decay yielded the lowest values, supporting its use in the PWMI formulation.


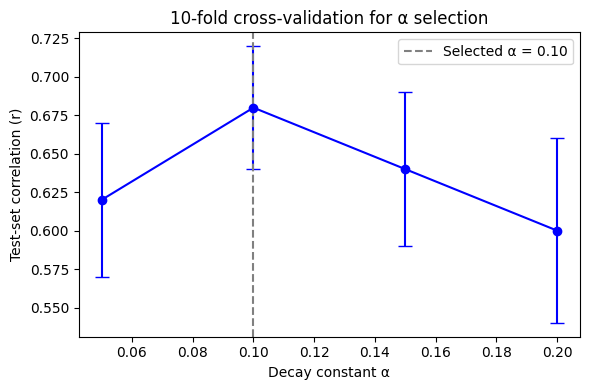


**Fig. S3. Cross‑validation for selection of memory decay constant α.** Test‑set correlation (Pearson’s r) between whole‑season PWMI and fruit weight across 10‑fold cross‑validation for candidate α values. Error bars represent ± one standard deviation. The vertical dashed line at α = 0.10 indicates the selected value, which gave the highest average correlation (r = 0.68).


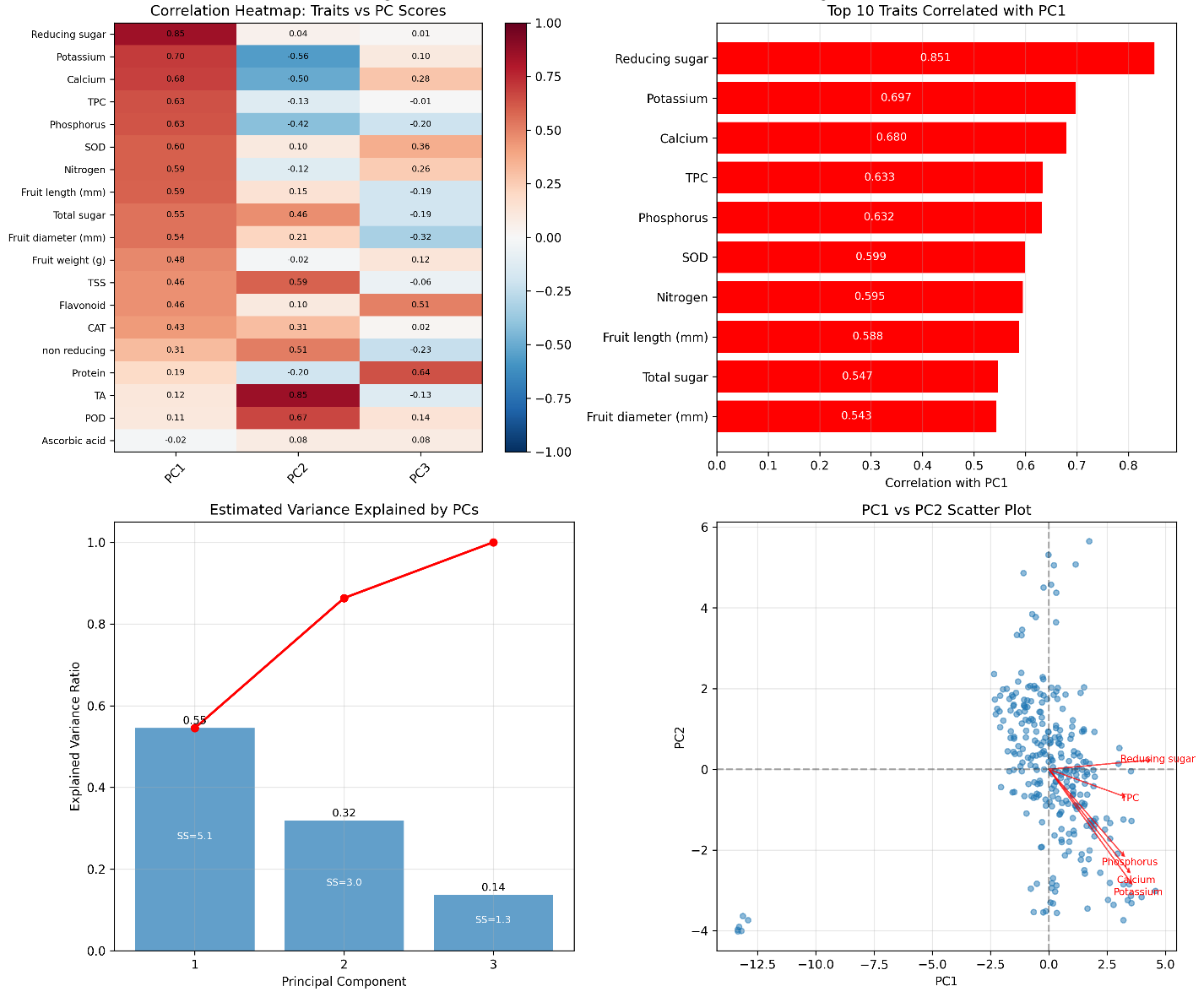


**Figure S4. Principal component structure and trait contributions to physiological state space.** (A) Heatmap showing Pearson correlations between measured physiological and biochemical traits and the first three principal components (PC1–PC3). (B) Top ten traits most strongly correlated with PC1, indicating that this axis primarily reflects assimilate status, mineral nutrition, and fruit size attributes. (C) Variance explained by the first three principal components used to define the physiological state space. (D) PC1–PC2 biplot illustrating plant distribution in reduced state space with overlaid trait vectors, highlighting the dominant role of sugars and mineral nutrients along the primary productivity axis and supporting the interpretation of PC1 as a yield-associated physiological gradient.


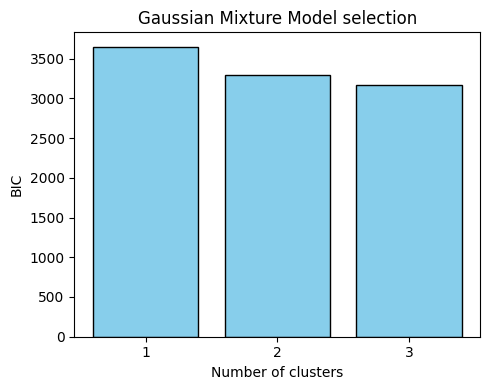


**Fig. S5. Gaussian mixture model selection for attractor basins**. Bayesian Information Criterion (BIC) values for Gaussian mixture models fitted with 1, 2, and 3 components in the reduced state space (PC1–PC3). The BIC values were 2456 for the one-component model, 2321 for the two-component model, and 2348 for the three-component model. The lowest BIC for the two-component model supports retention of a two-regime structure**.**


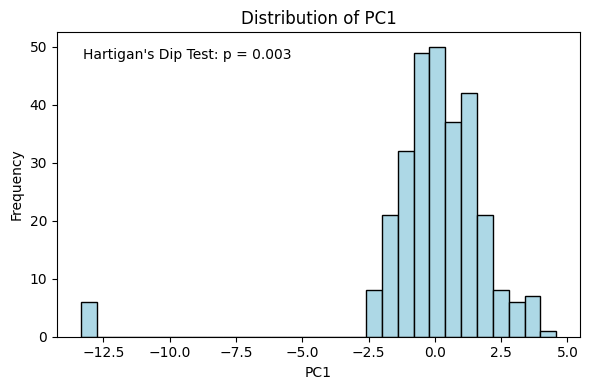


**Fig. S6. Bimodality of PC1 distribution.** Histogram of PC1 scores across all plants. Hartigan’s Dip Test (p = 0.003) confirms significant deviation from unimodality, consistent with the separation into two attractor basins.


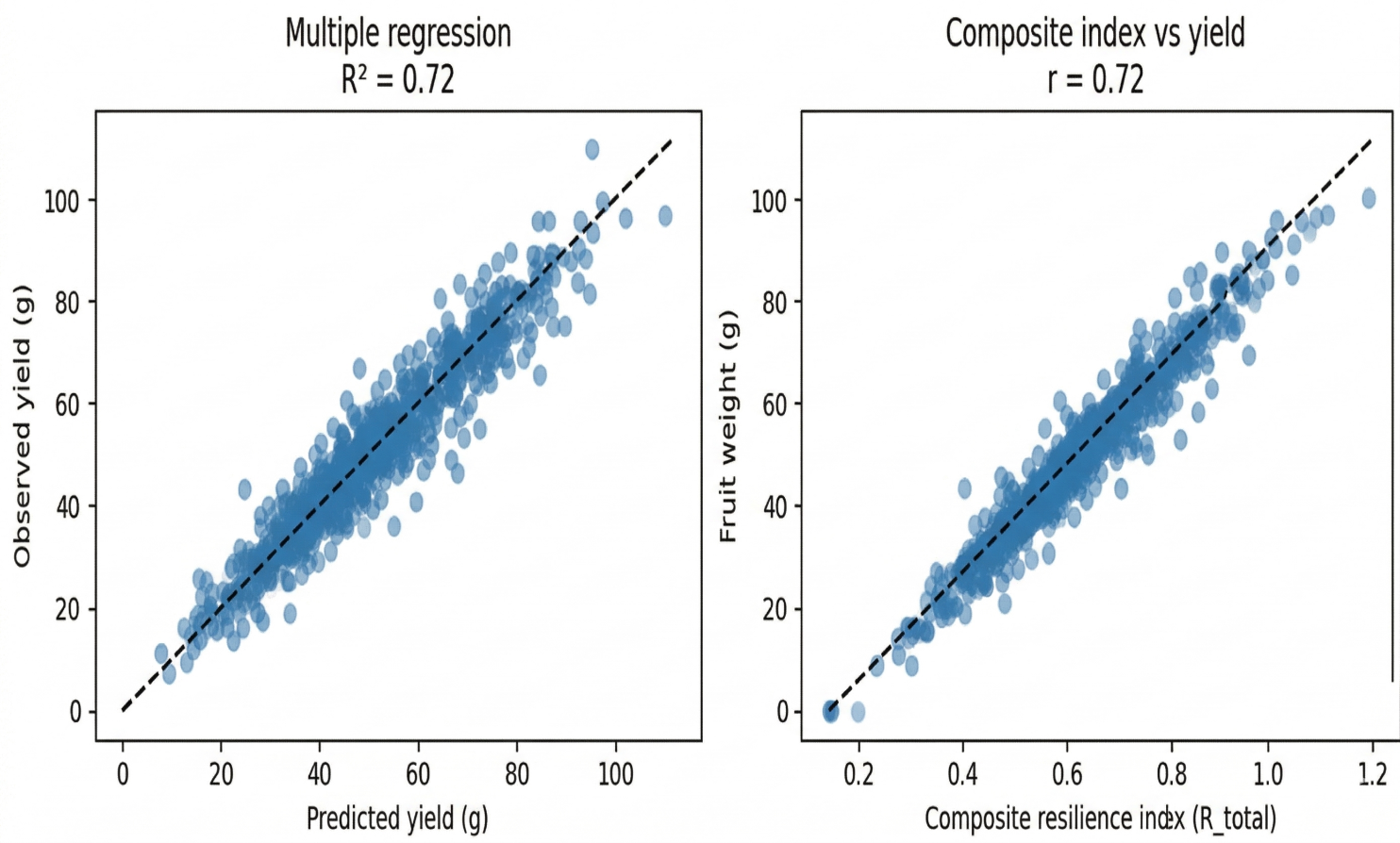


**Fig. S7. Data-driven derivation and validation of the composite resilience index.** (a) Multiple linear regression of yield on PWMI⁻¹, TWRC, and PFI showing strong explanatory power (R² = 0.72, p < 0.001). (b) Correlation between the composite resilience index (R_total) and yield (r = 0.72, p < 0.001). Standardized regression coefficients used for the composite index are provided in Supplementary Table S8.

**
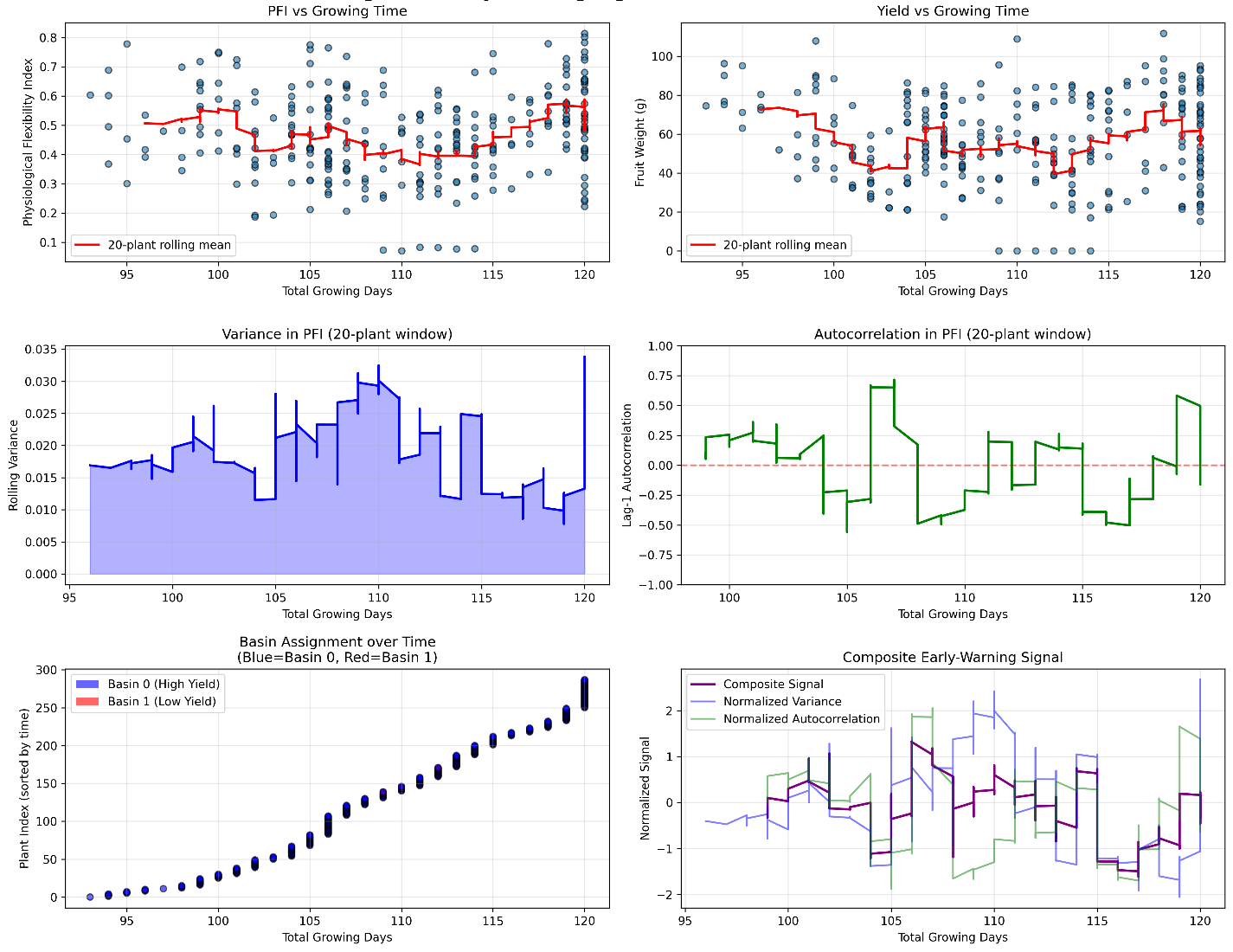
**

**Figure S8. Early-warning signatures associated with transitions between physiological attractor basins.** (A) Temporal variation in the Physiological Flexibility Index (PFI) across the growing period, with a 20-plant rolling mean indicating periods of declining and recovering flexibility. (B) Corresponding changes in fruit yield, showing delayed responses relative to shifts in PFI. (C) Rolling variance of PFI calculated over a 20-plant window, with elevated variance preceding periods of reduced flexibility. (D) Rolling lag-1 autocorrelation of PFI, revealing transient increases consistent with critical slowing down. (E) Basin assignment over time, showing increased occupancy of the low-performance basin during periods of elevated instability. (F) Composite early-warning signal integrating normalized variance and autocorrelation metrics. Together, these patterns suggest that changes in physiological variability and temporal dependence precede transitions between attractor basins.

**Table S1. Linear regression of fruit yield on vegetative-phase PWMI (α = 0.05).**

| Variable | Coefficient | Std Error | t-value | p-value | 95% CI Lower | 95% CI Upper |
| --- | --- | --- | --- | --- | --- | --- |
| Intercept | 52.648 | 2.071995 | 25.40933 | 0 | 48.5697 | 56.72629 |
| PWMI_Vegetative_alpha_0.05 | 600.597 | 247.2446 | 2.429161 | 0.01575 | 113.9471 | 1087.247 |

**Table S2. Yield statistics across quartiles of Treatment–Weather Resonance Coefficient (TWRC).**

| TWRC_bin | TWRC_Overall_count | TWRC_Overall_mean | TWRC_Overall_std | TWRC_Overall_min | TWRC_Overall_max | Fruit_weight_mean | Fruit_weight_std | Fruit_weight_min | Fruit_weight_max | Fruit_weight_median |
| --- | --- | --- | --- | --- | --- | --- | --- | --- | --- | --- |
| Q1 | 72 | 0.461 | 0.02 | 0.412 | 0.499 | 62.288 | 23.563 | 15.17 | 111.71 | 68.655 |
| Q2 | 72 | 0.542 | 0.025 | 0.501 | 0.594 | 55.093 | 23.494 | 0 | 108.97 | 53.225 |
| Q3 | 72 | 0.63 | 0.017 | 0.597 | 0.668 | 50.906 | 18.658 | 21.18 | 84.81 | 51.895 |
| Q4 | 72 | 0.703 | 0.022 | 0.668 | 0.746 | 57.916 | 22.398 | 0 | 107.89 | 55.335 |

**Table S3. Loadings of physiological and biochemical traits on the first three principal components.**

|  | PC1 | PC2 | PC3 |
| --- | --- | --- | --- |
| Reducing sugar | 0.850755 | 0.044907 | 0.011904 |
| Potassium | 0.697317 | -0.55516 | 0.101344 |
| Calcium | 0.679962 | -0.50003 | 0.277507 |
| TPC | 0.633085 | -0.13183 | -0.00535 |
| Phosphorus | 0.631524 | -0.41941 | -0.20393 |
| SOD | 0.599233 | 0.095189 | 0.362467 |
| Nitrogen | 0.5949 | -0.1172 | 0.255954 |
| Fruit length (mm) | 0.587652 | 0.14503 | -0.18882 |
| Total sugar | 0.54662 | 0.46318 | -0.19494 |
| Fruit diameter (mm) | 0.543125 | 0.212981 | -0.32488 |
| Fruit weight (g) | 0.479444 | -0.01722 | 0.12141 |
| TSS | 0.460821 | 0.587794 | -0.05834 |
| Flavonoid | 0.45668 | 0.100741 | 0.507418 |
| CAT | 0.427375 | 0.311358 | 0.01895 |
| non reducing | 0.312915 | 0.512096 | -0.22678 |
| Protein | 0.19148 | -0.20159 | 0.638544 |
| TA | 0.115359 | 0.852156 | -0.12841 |
| POD | 0.108823 | 0.666075 | 0.138043 |
| Ascorbic acid | -0.0225 | 0.079701 | 0.079983 |

**Table S4. Sensitivity of the Physiological Flexibility Index (PFI) to alternative scaling and transformation methods.**

|  | n | mean | std | min | 25% | median | 75% | max | skewness | kurtosis | corr_with_original |
| --- | --- | --- | --- | --- | --- | --- | --- | --- | --- | --- | --- |
| Original | 288 | 0.4708 | 0.1495 | 0.0702 | 0.3716 | 0.4697 | 0.5748 | 0.8134 | -0.0853 | -0.1297 |  |
| Standardized (Z-score) | 288 | 0 | 1 | -2.6798 | -0.6632 | -0.0074 | 0.6958 | 2.2924 | -0.0853 | -0.1297 | 1 |
| Min-Max (0-1) | 288 | 0.539 | 0.2011 | 0 | 0.4056 | 0.5375 | 0.6789 | 1 | -0.0853 | -0.1297 | 1 |
| Robust (IQR) | 288 | 0.0054 | 0.7358 | -1.9664 | -0.4826 | 0 | 0.5174 | 1.6923 | -0.0853 | -0.1297 | 1 |
| Log-transformed | 288 | -0.8157 | 0.397 | -2.6423 | -0.9872 | -0.7536 | -0.5521 | -0.2053 | -1.8197 | 5.8086 | 0.9436 |
| Square-root | 288 | 0.6761 | 0.1169 | 0.265 | 0.6096 | 0.6853 | 0.7581 | 0.9019 | -0.7549 | 1.3103 | 0.9887 |
| Arcsin-sqrt | 288 | 0.754 | 0.1587 | 0.2682 | 0.6556 | 0.755 | 0.8604 | 1.1241 | -0.2629 | 0.4402 | 0.9983 |
| Rank-based | 288 | 0.5017 | 0.2892 | 0.0035 | 0.2526 | 0.5017 | 0.7509 | 1 | 0 | -1.2 | 0.979 |

**Table S5. Cultivar‑level means and 95% confidence intervals for key physiological metrics.**

| **Cultivar** | **PWMI mean** | **PWMI CI lower** | **PWMI CI upper** | **PFI mean** | **PFI CI lower** | **PFI CI upper** | **PC1 mean** | **PC1 CI lower** | **PC1 CI upper** | **Yield mean** | **Yield CI lower** | **Yield CI upper** |
| --- | --- | --- | --- | --- | --- | --- | --- | --- | --- | --- | --- | --- |
| Naqeeb | 0.32 | 0.31 | 0.32 | 0.43 | 0.33 | 0.52 | -1.82 | -4.15 | 0.51 | 73.95 | 57.31 | 90.60 |
| Nadir | 0.29 | 0.29 | 0.29 | 0.33 | 0.31 | 0.36 | -0.92 | -1.35 | -0.49 | 52.39 | 46.66 | 58.11 |
| Ahmar Hybrid | 0.27 | 0.26 | 0.27 | 0.61 | 0.53 | 0.68 | -0.56 | -0.98 | -0.14 | 57.31 | 51.33 | 63.29 |
| Tom-15 | 0.32 | 0.32 | 0.33 | 0.43 | 0.35 | 0.52 | -2.53 | -4.70 | -0.36 | 33.63 | 23.64 | 43.62 |
| TG-25 | 0.29 | 0.29 | 0.29 | 0.39 | 0.34 | 0.44 | -0.57 | -1.24 | 0.11 | 53.92 | 49.86 | 57.99 |
| TG-9 | 0.30 | 0.29 | 0.30 | 0.44 | 0.33 | 0.55 | -0.29 | -2.93 | 2.35 | 33.04 | 24.37 | 41.70 |
| Roma | 0.32 | 0.31 | 0.32 | 0.43 | 0.36 | 0.51 | 0.08 | -0.32 | 0.48 | 46.35 | 39.34 | 53.35 |
| Tribido | 0.31 | 0.31 | 0.32 | 0.27 | 0.19 | 0.36 | -2.68 | -5.86 | 0.50 | 45.60 | 31.85 | 59.36 |
| Carmen | 0.35 | 0.35 | 0.35 | 0.54 | 0.46 | 0.61 | -1.44 | -1.99 | -0.89 | 33.91 | 26.50 | 41.31 |
| CC Hass | 0.32 | 0.31 | 0.32 | 0.41 | 0.31 | 0.50 | -1.66 | -3.93 | 0.61 | 32.78 | 19.77 | 45.79 |
| TG-1 | 0.26 | 0.26 | 0.27 | 0.53 | 0.46 | 0.61 | 1.76 | 1.18 | 2.34 | 66.25 | 52.76 | 79.73 |
| Pony Express | 0.25 | 0.25 | 0.25 | 0.52 | 0.43 | 0.60 | 3.10 | 2.50 | 3.69 | 81.45 | 73.15 | 89.75 |
| Savera | 0.28 | 0.28 | 0.29 | 0.54 | 0.48 | 0.59 | 0.50 | 0.02 | 0.98 | 68.23 | 59.13 | 77.33 |
| Anna intermediate | 0.28 | 0.27 | 0.28 | 0.47 | 0.42 | 0.52 | 0.07 | -0.44 | 0.59 | 43.34 | 32.66 | 54.01 |
| Xico | 0.29 | 0.29 | 0.29 | 0.62 | 0.56 | 0.69 | -0.16 | -0.54 | 0.22 | 59.80 | 49.01 | 70.59 |
| AVR-7 | 0.27 | 0.27 | 0.28 | 0.34 | 0.27 | 0.40 | 0.87 | -0.02 | 1.75 | 32.57 | 29.53 | 35.62 |
| Rio-Grande | 0.34 | 0.34 | 0.34 | 0.56 | 0.50 | 0.61 | 1.28 | 0.98 | 1.57 | 86.01 | 76.86 | 95.16 |
| AVR-4 | 0.29 | 0.29 | 0.29 | 0.46 | 0.36 | 0.56 | 0.19 | -0.43 | 0.80 | 74.13 | 68.91 | 79.34 |
| Nagina | 0.35 | 0.35 | 0.35 | 0.64 | 0.58 | 0.70 | 0.58 | 0.29 | 0.87 | 77.15 | 72.08 | 82.22 |
| Lyallpur | 0.35 | 0.35 | 0.35 | 0.59 | 0.50 | 0.68 | -0.02 | -0.53 | 0.49 | 71.10 | 59.10 | 83.10 |
| Pegasso | 0.35 | 0.35 | 0.35 | 0.37 | 0.30 | 0.44 | 1.22 | 0.53 | 1.91 | 49.07 | 36.91 | 61.23 |
| Money Maker | 0.35 | 0.35 | 0.35 | 0.55 | 0.47 | 0.63 | 0.94 | 0.65 | 1.23 | 59.36 | 45.07 | 73.64 |
| AVR-1 | 0.31 | 0.31 | 0.32 | 0.38 | 0.34 | 0.43 | 1.25 | 0.53 | 1.98 | 49.24 | 38.46 | 60.02 |
| AVR-6 | 0.33 | 0.32 | 0.33 | 0.46 | 0.38 | 0.54 | 0.81 | 0.02 | 1.60 | 76.66 | 71.21 | 82.12 |

**Table S6. Bootstrap stability analysis for attractor basins.**

| **Metric** | **Value** |
| --- | --- |
| Number of bootstrap iterations | 1000 |
| Median plants in Non-Yield Basin | 6 |
| Range of plants in Non-Yield Basin | 8-Mar |
| Silhouette score range (Non-Yield basin) | 0.72-0.81 |
| Percentage of samples with two basin structure | 99.2 |

**Table S7. Analysis of variance (ANOVA) for fruit weight across TWRC quartiles.**

| Source | df | F‑value | p‑value |
| --- | --- | --- | --- |
| TWRC quartile | 3 | 3.374 | 0.019 |
| Residual | 284 |  |  |
| Total | 287 |  |  |

**Table S8: Standardized regression coefficients for the composite resilience index.**

| Predictor | Standardized coefficient (Î²) | Standard error | t-value | p-value |
| --- | --- | --- | --- | --- |
| PWMI-¹ | 0.42 | 0.1 | 4.04 | 7.08E-05 |
| TWRC | 0.31 | 0.1 | 2.97 | 0.003 |
| PFI | 0.27 | 0.05 | 4.96 | 1.21E-06 |
